# Association between body roundness index and pregnancy outcomes of IVF

**DOI:** 10.1093/hropen/hoag068

**Published:** 2026-08-04

**Authors:** Yuan Fang, Yufang Yang, Ning Li, Jialin Zou, Yue Niu, Dingying Zhao, Sirui Liao, Yanran Liu, Zi-Jiang Chen, Daimin Wei

**Affiliations:** State Key Laboratory of Reproductive Medicine and Offspring Health, Center for Reproductive Medicine, Institute of Women, Children and Reproductive Health, Shandong University, Jinan, Shandong, China; National Research Center for Assisted Reproductive Technology and Reproductive Genetics, Shandong University, Jinan, Shandong, China; Ministry of Education, Key Laboratory of Reproductive Endocrinology (Shandong University), Jinan, Shandong, China; Shandong Technology Innovation Center for Reproductive Health, Jinan, Shandong, China; Shandong Provincial Clinical Research Center for Reproductive Health, Jinan, Shandong, China; Shandong Key Laboratory of Reproductive Research and Birth Defect Prevention, Jinan, Shandong, China; Research Unit of Gametogenesis and Health of ART-Offspring, Chinese Academy of Medical Sciences (No. 2021RU001), Jinan, Shandong, China; State Key Laboratory of Reproductive Medicine and Offspring Health, Center for Reproductive Medicine, Institute of Women, Children and Reproductive Health, Shandong University, Jinan, Shandong, China; National Research Center for Assisted Reproductive Technology and Reproductive Genetics, Shandong University, Jinan, Shandong, China; Ministry of Education, Key Laboratory of Reproductive Endocrinology (Shandong University), Jinan, Shandong, China; Shandong Technology Innovation Center for Reproductive Health, Jinan, Shandong, China; Shandong Provincial Clinical Research Center for Reproductive Health, Jinan, Shandong, China; Shandong Key Laboratory of Reproductive Research and Birth Defect Prevention, Jinan, Shandong, China; Research Unit of Gametogenesis and Health of ART-Offspring, Chinese Academy of Medical Sciences (No. 2021RU001), Jinan, Shandong, China; State Key Laboratory of Reproductive Medicine and Offspring Health, Center for Reproductive Medicine, Institute of Women, Children and Reproductive Health, Shandong University, Jinan, Shandong, China; National Research Center for Assisted Reproductive Technology and Reproductive Genetics, Shandong University, Jinan, Shandong, China; Ministry of Education, Key Laboratory of Reproductive Endocrinology (Shandong University), Jinan, Shandong, China; Shandong Technology Innovation Center for Reproductive Health, Jinan, Shandong, China; Shandong Provincial Clinical Research Center for Reproductive Health, Jinan, Shandong, China; Shandong Key Laboratory of Reproductive Research and Birth Defect Prevention, Jinan, Shandong, China; Research Unit of Gametogenesis and Health of ART-Offspring, Chinese Academy of Medical Sciences (No. 2021RU001), Jinan, Shandong, China; State Key Laboratory of Reproductive Medicine and Offspring Health, Center for Reproductive Medicine, Institute of Women, Children and Reproductive Health, Shandong University, Jinan, Shandong, China; National Research Center for Assisted Reproductive Technology and Reproductive Genetics, Shandong University, Jinan, Shandong, China; Ministry of Education, Key Laboratory of Reproductive Endocrinology (Shandong University), Jinan, Shandong, China; Shandong Technology Innovation Center for Reproductive Health, Jinan, Shandong, China; Shandong Provincial Clinical Research Center for Reproductive Health, Jinan, Shandong, China; Shandong Key Laboratory of Reproductive Research and Birth Defect Prevention, Jinan, Shandong, China; Research Unit of Gametogenesis and Health of ART-Offspring, Chinese Academy of Medical Sciences (No. 2021RU001), Jinan, Shandong, China; State Key Laboratory of Reproductive Medicine and Offspring Health, Center for Reproductive Medicine, Institute of Women, Children and Reproductive Health, Shandong University, Jinan, Shandong, China; National Research Center for Assisted Reproductive Technology and Reproductive Genetics, Shandong University, Jinan, Shandong, China; Ministry of Education, Key Laboratory of Reproductive Endocrinology (Shandong University), Jinan, Shandong, China; Shandong Technology Innovation Center for Reproductive Health, Jinan, Shandong, China; Shandong Provincial Clinical Research Center for Reproductive Health, Jinan, Shandong, China; Shandong Key Laboratory of Reproductive Research and Birth Defect Prevention, Jinan, Shandong, China; Research Unit of Gametogenesis and Health of ART-Offspring, Chinese Academy of Medical Sciences (No. 2021RU001), Jinan, Shandong, China; State Key Laboratory of Reproductive Medicine and Offspring Health, Center for Reproductive Medicine, Institute of Women, Children and Reproductive Health, Shandong University, Jinan, Shandong, China; National Research Center for Assisted Reproductive Technology and Reproductive Genetics, Shandong University, Jinan, Shandong, China; Ministry of Education, Key Laboratory of Reproductive Endocrinology (Shandong University), Jinan, Shandong, China; Shandong Technology Innovation Center for Reproductive Health, Jinan, Shandong, China; Shandong Provincial Clinical Research Center for Reproductive Health, Jinan, Shandong, China; Shandong Key Laboratory of Reproductive Research and Birth Defect Prevention, Jinan, Shandong, China; Research Unit of Gametogenesis and Health of ART-Offspring, Chinese Academy of Medical Sciences (No. 2021RU001), Jinan, Shandong, China; State Key Laboratory of Reproductive Medicine and Offspring Health, Center for Reproductive Medicine, Institute of Women, Children and Reproductive Health, Shandong University, Jinan, Shandong, China; National Research Center for Assisted Reproductive Technology and Reproductive Genetics, Shandong University, Jinan, Shandong, China; Ministry of Education, Key Laboratory of Reproductive Endocrinology (Shandong University), Jinan, Shandong, China; Shandong Technology Innovation Center for Reproductive Health, Jinan, Shandong, China; Shandong Provincial Clinical Research Center for Reproductive Health, Jinan, Shandong, China; Shandong Key Laboratory of Reproductive Research and Birth Defect Prevention, Jinan, Shandong, China; Research Unit of Gametogenesis and Health of ART-Offspring, Chinese Academy of Medical Sciences (No. 2021RU001), Jinan, Shandong, China; State Key Laboratory of Reproductive Medicine and Offspring Health, Center for Reproductive Medicine, Institute of Women, Children and Reproductive Health, Shandong University, Jinan, Shandong, China; National Research Center for Assisted Reproductive Technology and Reproductive Genetics, Shandong University, Jinan, Shandong, China; Ministry of Education, Key Laboratory of Reproductive Endocrinology (Shandong University), Jinan, Shandong, China; Shandong Technology Innovation Center for Reproductive Health, Jinan, Shandong, China; Shandong Provincial Clinical Research Center for Reproductive Health, Jinan, Shandong, China; Shandong Key Laboratory of Reproductive Research and Birth Defect Prevention, Jinan, Shandong, China; Research Unit of Gametogenesis and Health of ART-Offspring, Chinese Academy of Medical Sciences (No. 2021RU001), Jinan, Shandong, China; State Key Laboratory of Reproductive Medicine and Offspring Health, Center for Reproductive Medicine, Institute of Women, Children and Reproductive Health, Shandong University, Jinan, Shandong, China; National Research Center for Assisted Reproductive Technology and Reproductive Genetics, Shandong University, Jinan, Shandong, China; Ministry of Education, Key Laboratory of Reproductive Endocrinology (Shandong University), Jinan, Shandong, China; Shandong Technology Innovation Center for Reproductive Health, Jinan, Shandong, China; Shandong Provincial Clinical Research Center for Reproductive Health, Jinan, Shandong, China; Shandong Key Laboratory of Reproductive Research and Birth Defect Prevention, Jinan, Shandong, China; Research Unit of Gametogenesis and Health of ART-Offspring, Chinese Academy of Medical Sciences (No. 2021RU001), Jinan, Shandong, China; Shanghai Key Laboratory for Assisted Reproduction and Reproductive Genetics, Shanghai, China; Department of Reproductive Medicine, Ren Ji Hospital, Shanghai Jiao Tong University School of Medicine, Shanghai, China; State Key Laboratory of Reproductive Medicine and Offspring Health, Center for Reproductive Medicine, Institute of Women, Children and Reproductive Health, Shandong University, Jinan, Shandong, China; National Research Center for Assisted Reproductive Technology and Reproductive Genetics, Shandong University, Jinan, Shandong, China; Ministry of Education, Key Laboratory of Reproductive Endocrinology (Shandong University), Jinan, Shandong, China; Shandong Technology Innovation Center for Reproductive Health, Jinan, Shandong, China; Shandong Provincial Clinical Research Center for Reproductive Health, Jinan, Shandong, China; Shandong Key Laboratory of Reproductive Research and Birth Defect Prevention, Jinan, Shandong, China; Research Unit of Gametogenesis and Health of ART-Offspring, Chinese Academy of Medical Sciences (No. 2021RU001), Jinan, Shandong, China

**Keywords:** BMI, body roundness index, IVF, pregnancy outcomes, live birth

## Abstract

**STUDY QUESTION:**

Is there an association between the body roundness index (BRI) and pregnancy outcomes of IVF, and can it provide complementary information beyond body mass index (BMI)?

**SUMMARY ANSWER:**

Higher BRI was associated with a lower likelihood of live birth following IVF, particularly among women with normal BMI.

**WHAT IS KNOWN ALREADY:**

BMI is widely used to assess obesity-related reproductive risk but does not adequately characterize body fat distribution. BRI, a novel anthropometric index that may better reflect central adiposity, has been associated with cardiometabolic risk, but its relationship with IVF outcomes remains unclear.

**STUDY DESIGN, SIZE, DURATION:**

This study was a secondary analysis of data from two multicentre randomized controlled trials (RCTs) in China that evaluated live birth rates between freeze-only and fresh embryo transfers. The first trial enrolled 1508 women with polycystic ovary syndrome from 14 centres between June 2013 and May 2014, with follow-up completed in July 2015. The second trial recruited 2157 women with regular menstrual cycles from 20 centres between March 2015 and November 2015, with follow-up completed in March 2017.

**PARTICIPANTS/MATERIALS, SETTING, METHODS:**

After exclusions, 3523 women who underwent embryo transfer and had complete BRI data were included in the analysis. BRI was calculated from height and waist circumference measured prior to IVF. In the absence of universally accepted clinical thresholds for BRI, the participants were categorized into three groups according to the 33rd and 66th percentiles: <2.54 (n = 1171), 2.54 to <3.54 (n = 1174), ≥3.54 (n = 1178). The primary outcome was live birth, which was defined as the delivery of neonate(s) with signs of life at ≥28 weeks’ gestation. Modified Poisson regression with robust variance was used to estimate adjusted risk ratios (aRRs) and 95% confidence intervals (CIs). The models were adjusted for age (continuous), duration of infertility (continuous), cause of infertility, type of embryo transfer, diagnosis of polycystic ovary syndrome (PCOS), number of oocytes retrieved (continuous), and number of embryo(s) transferred. These covariates were selected based on clinical relevance and prior literature to reduce potential confounding.

**MAIN RESULTS AND THE ROLE OF CHANCE:**

The rate of live birth was lower among women in the highest BRI group (group 3) compared with those in the lowest group (group 1) (45.2% vs 53.6%; aRR, 0.89; 95% confidence interval [CI], 0.81–0.97; *P *= 0.008). Women in group 3 also had an increased risk of total pregnancy loss compared with those in group 1 (aRR, 1.31; 95% CI, 1.07–1.61; *P *= 0.009). In BMI-stratified analyses, higher BRI was associated with a reduced likelihood of live birth (group 2, aRR, 0.89; 95% CI, 0.81–0.98; *P *= 0.019; group 3, aRR, 0.84; 95% CI, 0.73–0.96; *P *= 0.009) among women with normal BMI (18.5 to <24 kg/m^2^). Furthermore, each one-unit increase in BRI was associated with a 7% lower likelihood of live birth (aRR, 0.93; 95% CI, 0.87–0.98; *P *= 0.009) among women with normal BMI. No significant association between BRI and live birth was observed among women who were overweight or obese (BMI ≥ 24 kg/m^2^).

**LIMITATIONS, REASONS FOR CAUTION:**

In this post-hoc analysis, causality cannot be established, and residual confounding cannot be ruled out. The small-sized subgroups, particularly among women with overweight or obesity, may have limited statistical power.

**WIDER IMPLICATIONS OF THE FINDINGS:**

These findings suggest that BRI may capture adiposity-related risk not reflected by BMI alone, particularly among women with normal BMI. BRI may serve as a complementary anthropometric measure alongside BMI for identifying women at higher risk of adverse IVF outcomes. Further studies are needed to validate these findings across BMI categories and clarify the clinical utility of BRI in IVF populations.

**FUNDING:**

This study was supported by the National Key Research and Development Program of China (2023YFC2705502) and the National Natural Science Foundation of China (82495194 and 82421004).

**DISCLOSURES:**

There are no conflicts of interest to declare.

**TRIAL REGISTRATION NUMBER:**

N/A.

WHAT DOES THIS MEAN FOR PATIENTS? Body mass index (BMI) is commonly used to assess whether obesity may affect health. However, BMI does not adequately capture body fat distribution, particularly ‘central adiposity’—some women with normal BMI may still carry more fat around the waist and internal organs. The body roundness index (BRI), which is calculated from height and waist circumference, may provide additional information about fat distribution. Higher BRI values generally indicate greater central adiposity. However, there is currently no universally accepted range defining which BRI values are healthy or harmful.

This study included 3523 women undergoing IVF treatment and investigated whether BRI was linked to pregnancy outcomes. We found that women with a higher BRI were less likely to have a live birth and were more likely to experience pregnancy loss than women with a lower BRI, particularly among women with normal BMI. These findings suggest that BRI may provide additional information beyond BMI. Further research is needed to determine whether BRI could help predict adverse pregnancy outcomes.

## Introduction

Obesity is a highly prevalent chronic disease worldwide that affects 40% of reproductive-aged females (Schon *et al.*, 2024). Body mass index (BMI), a traditional obesity metric, has been widely used to assess the impact of obesity on reproductive health. Generally, higher BMI is related to an increased risk for female infertility, and many women with higher BMI subsequently seek IVF treatment (Sermondade *et al.*, 2019). Increasing observational evidence has suggested that higher BMI among women is associated with poorer IVF outcomes, including higher cycle cancellation rates, increased risk of miscarriage and, consequently, lower live birth rates (Sermondade *et al.*, 2019; Cozzolino *et al.*, 2021; Fabozzi *et al.*, 2024; Ren *et al.*, 2025). Despite its widespread clinical use, BMI has recognized limitations. Because BMI does not distinguish between fat mass and lean mass, it may misclassify individuals with high muscle mass as overweight or obese (Darici *et al.*, 2025; Hegazi and Halpern, 2025). More importantly, BMI does not adequately capture body fat distribution, particularly central or visceral adiposity (Du *et al.*, 2013; Schweitzer, 2024; Darici *et al.*, 2025). Consequently, individuals with similar BMI may differ substantially in body composition, metabolic health, and reproductive outcomes. For example, women with normal BMI may still exhibit excess central adiposity and metabolic abnormalities (Schweitzer, 2024). Emerging evidence has suggested that excess visceral fat, rather than BMI alone, may better predict metabolic dysfunction and poor reproductive outcomes (Darici *et al.*, 2025; Rubino *et al.*, 2025).

The body roundness index (BRI), a novel metric calculated from height and waist circumference, is considered a better estimate of central adiposity than BMI (Thomas *et al.*, 2013). BRI may help identify individuals with excess central adiposity despite having normal or relatively low BMIs. Several studies found that BRI was superior to other anthropometric indicators in estimating the risk of metabolic-related diseases including diabetes and cardiovascular disease (Rico-Martín *et al.*, 2020; Wu *et al.*, 2022; Cai *et al.*, 2023). Unlike BMI, BRI currently has no universally accepted clinical thresholds, and reported thresholds may vary across populations and outcomes. Moreover, there is a paucity of evidence regarding the association between BRI and reproductive outcomes and about whether BRI offers additional information beyond BMI.

In this study of women undergoing IVF treatment, we investigated the association between female BRI and pregnancy outcomes. In addition, we explored whether BRI could identify risk heterogeneity not captured by BMI, particularly among women with normal BMI.

## Materials and methods

This study is a secondary analysis of data from two multicentre randomized controlled trials (RCTs) that evaluated the live birth rates between freeze-only and fresh embryo transfer. The original randomized controlled trials were conducted by Shandong University and other reproductive centres in China, and the relevant study protocols were published (Chen *et al.*, 2016; Shi *et al.*, 2018). The first trial enrolled 1508 women with polycystic ovary syndrome (PCOS) from 14 centres between June 2013 and May 2014, with follow-up completed in July 2015 (registration numbers: NCT01841528). The second trial recruited 2157 women with regular menstrual cycles from 20 centres between March 2015 and November 2015, with follow-up completed in March 2017 (registration number: ChiCTR-IOR-14005406). The two RCTs used largely similar IVF treatment protocols and laboratory procedures. The main difference between the two trials was the enrolled populations and the regimens for endometrial preparation and luteal phase support. Both the original study and this study were approved by the ethics committees of the Reproductive Medicine Center of Shandong University (Ethics Approval Number: [2012] Lun Shen Zi No. (15), [2014] Lun Shen Zi No. (18), [2025] Lun Shen Zi No. (185)). The report of findings followed Strengthening the Reporting of Observational Studies in Epidemiology (STROBE) guidelines.

### Study population

The original two RCTs included 3665 women with PCOS or regular menstrual cycles who were undergoing their first cycle of IVF. Exclusion criteria were: (i) women who did not undergo embryo transfer and (ii) women with incomplete BRI calculation data.

### IVF procedure

A gonadotropin-releasing–hormone (GnRH) antagonist protocol was used for ovarian stimulation in all women, with subsequent oocyte retrieval and fertilization. On the day of oocyte retrieval, the women were randomized to either the fresh embryo transfer group or the frozen embryo transfer group. Either a single blastocyst-stage embryo or up to two cleavage-stage embryos were transferred. For women in the fresh embryo group, luteal phase support was initiated on the day of oocyte retrieval. For women in the frozen embryo group, all embryos were vitrified, and the endometrium was prepared using a natural ovulatory or programmed regimen. Two weeks after transfer, the serum level of human chorionic gonadotropin (hCG) was measured. For the women who achieved pregnancy, luteal phase support was continued until 10 weeks of gestation and all pregnancies were followed until delivery or pregnancy loss. Details of the studies have previously been described (Chen *et al.*, 2016; Shi *et al.*, 2018).

### Exposures

All body measurement values, including height, weight, waist circumference (WC), and blood pressure (BP, including SBP (systolic blood pressure) and DBP (diastolic blood pressure)) were conducted by trained staff. The BRI is calculated as follows:


$$\mathrm{BRI}=364.2-365.5\times \sqrt{1-{\left(\frac{\mathrm{WC}}{\pi \times \mathrm{height}}\right)}^{2}}$$

WC and height were expressed in the same units. Women were categorized into three groups according to the quantiles of BRI (33rd and 66th percentiles): group 1 (BRI < 2.54), group 2 (2.54 ≤ BRI < 3.54), and group 3 (BRI ≥ 3.54), with group 1 serving as the reference group.

### Outcomes

The primary outcome was live birth after the first embryo transfer, which was defined as the delivery of neonate(s) with signs of life at ≥28 weeks’ gestation. The secondary outcomes included biochemical pregnancy (defined as serum hCG level ≥10 IU/l after embryo transfer), clinical pregnancy (defined as ultrasound confirmation of at least 1 intrauterine gestational sac 30–35 days after embryo transfer), ongoing pregnancy (defined as detection of a viable fetus with fetal heartbeat at 11–12 weeks’ gestation), total pregnancy loss (included both biochemical pregnancy loss and clinical pregnancy loss), biochemical pregnancy loss (defined as the absence of an identifiable pregnancy on ultrasound examination despite a positive hCG pregnancy test), and clinical pregnancy loss (defined as miscarriage or therapeutic abortion of a clinical pregnancy before 28 weeks of gestation).

### Statistical analysis

Continuous variables following or approximating a normal distribution were expressed as mean ± standard deviation (SD) and compared using ANOVA. Continuous variables that did not follow a normal distribution were expressed as median and interquartile range (IQR) and compared using Kruskal–Wallis tests. Categorical variables were summarized as frequencies (percentages) and analyzed using Pearson’s chi-squared test or Fisher’s exact test, as appropriate.

Modified Poisson regression with robust variance was used to estimate adjusted risk ratios (aRRs) and 95% confidence intervals (CIs) for the associations between BRI and pregnancy outcomes because it is less prone to convergence problems and could directly estimate relative risk (Zou, 2004; Chen *et al.*, 2018). Covariates were selected based on clinical relevance and evidence from previous literature. The primary multivariable model was adjusted for age (continuous), duration of infertility (continuous), cause of infertility, type of embryo transfer, diagnosis of PCOS, number of oocytes retrieved (continuous), and number of embryo(s) transferred. BRI was also modeled as a continuous variable, and its average effects on pregnancy outcomes were estimated per one-unit increment in BRI. We utilized restricted cubic spline (RCS) models based on modified Poisson regression (3 knots at the 10th, 50th, and 90th percentiles) to explore the potential non-linear relationships between BRI and pregnancy outcomes. These RCS models used the same covariate adjustment as the primary multivariable model. The nonlinearity was assessed using Wald *χ*² tests. To examine whether the association between BRI and pregnancy outcomes differed by BMI category, we performed BMI-stratified analyses among women with normal BMI (18.5 to <24.0 kg/m²) and women who were overweight or obese (BMI ≥24.0 kg/m²). Women who were underweight (BMI <18.5 kg/m²) were not analyzed as a separate BMI stratum because of the small subgroup size. Interaction terms between BRI and BMI group were additionally included to test for effect modification. Subsequently, Pearson and Spearman correlation coefficients were calculated to assess the correlation between continuous BRI and BMI in the overall population and within BMI strata. To evaluate whether BRI provided information beyond BMI, sensitivity analyses were performed by further adjusting for BMI based on the primary multivariable model. Multicollinearity was assessed using variance inflation factors (VIF), or generalized variance inflation factors (GVIF), as appropriate.

In addition, we conducted subgroup analyses by maternal age, type of embryo transfer, and cause of infertility, and the results were presented as forest plots. Additional stratified analyses were conducted according to PCOS status, and the interaction between BRI and PCOS status was tested. A sensitivity analysis was performed after excluding women with underweight. To assess the potential influence of ovarian reserve and ovarian response indicators, three additional models were constructed based on the primary multivariable model (Model 1). Model 2 was additionally adjusted for antral follicle count (AFC) and anti-Müllerian hormone (AMH). Model 3 was additionally adjusted for duration of ovarian stimulation, gonadotropin dose, and estradiol level on the day of hCG trigger. Model 4 was additionally adjusted for all five variables.

A two-sided *P *< 0.05 was considered statistically significant for the main outcome of live birth. Analyses of secondary outcomes were exploratory because no adjustments were made for multiple comparisons. All statistical analyses were performed using R software (version 4.4.2; Vienna, Austria).

## Results

### Baseline characteristics

**Figure 1.**
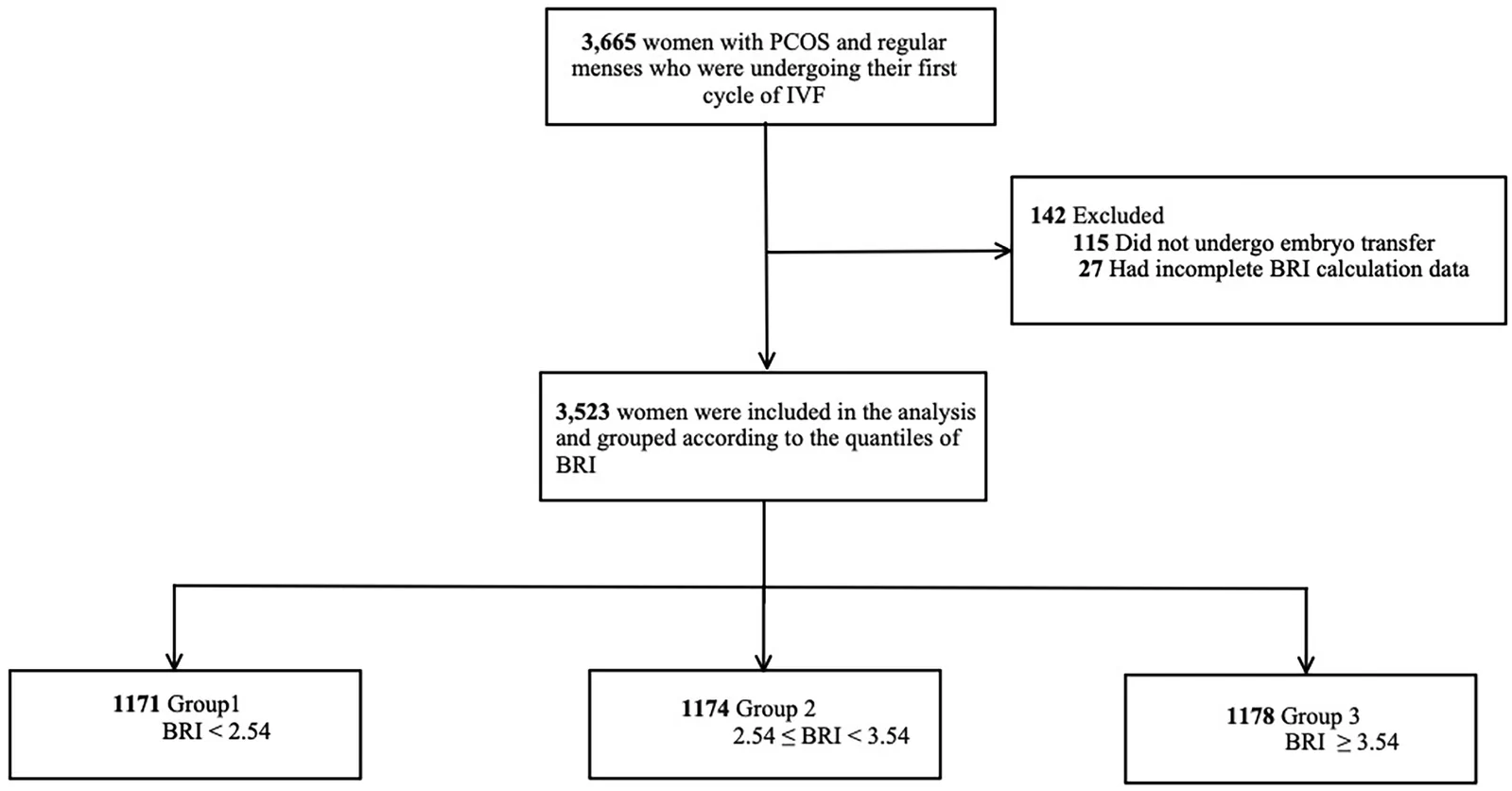
**Study flow chart**. Flow diagram illustrating participant selection, inclusion, and analysis among women undergoing IVF. PCOS, polycystic ovary syndrome; BRI, body roundness index.

A total of 3523 eligible women were included in the analysis (Fig. 1). The baseline characteristics of women are presented in Table 1. There were 1171 women in group 1, 1174 women in group 2, and 1178 women in group 3. The minor differences in group sizes across BRI tertiles were expected, as the total sample size (n = 3523) was not evenly divisible by three, and tied values of BRI at cut points may affect group allocation. The mean (SD) age was 28.3 [2.0] years, and the mean BMI was 22.8 [3.4] kg/m^2^. Compared to women in group 1, women in group 2 or 3 had higher BMIs (mean [SD] 20.3 [2.2] kg/m^2^ vs 22.4 [2.3] kg/m^2^ vs 25.7 [3.2] kg/m^2^) and were also more likely to be diagnosed with PCOS (17.2% vs 42.2% vs 65.7%) (*P *< 0.001) (Table 1).

**Table 1.** Demographic and clinical characteristics of participants according to tertiles of body roundness index.

Characteristic	BRI group 1 (<2.54) (n = 1171)	BRI group 2 (2.54 to <3.54) (n = 1174)	BRI group 3 (≥3.54) (n = 1178)	*P-*value
Age, mean (SD), years	28.1 (3.0)	28.3 (3.0)	28.6 (3.1)	**<0.001** ***
Cause of infertility				**0.004** **
Tubal factor	680 (58.1)	689 (58.7)	697 (59.2)	
Male factor	313 (26.7)	277 (23.6)	248 (21.1)	
Others	178 (15.2)	208 (17.7)	233 (19.8)	
Duration of infertility, median (IQR), years	3.0 (2.0–4.0)	3.0 (2.0–4.4)	3.5 (2.0–5.0)	**0.001** **
Type of embryo transfer				**0.026** *
Fresh embryo transfer	556 (47.5)	623 (53.1)	595 (50.5)	
Frozen embryo transfer	615 (52.5)	551 (46.9)	583 (49.5)	
PCOS diagnosis	201 (17.2)	496 (42.2)	774 (65.7)	**<0.001** ***
AFC, median (IQR)	16.0 (12.0–22.0)	20.0 (14.0–26.0)	24.0 (17.0–30.0)	**<0.001** ***
AMH, median (IQR), ng/ml	4.8 (3.0–7.1)	5.0 (3.2–8.4)	5.0 (3.2–8.5)	**0.036** *
No. of oocytes retrieved, median (IQR)	12.0 (9.0–16.0)	13.0 (9.0–16.8)	13.0 (9.00–17.0)	**0.007** **
Number of embryo(s) transferred				0.232
One	85 (7.3)	71 (6.0)	66 (5.6)	
Two	1086 (92.7)	1103 (94.0)	1112 (94.4)	
Stage of embryo transferred				0.128
Cleavage	1103 (94.2)	1127 (96.0)	1121 (95.2)	
Blastocyst	68 (5.8)	47 (4.0)	57 (4.8)	
Duration of ovarian stimulation, median (IQR), days	9.0 (9.0–10.0)	10.0 (9.0–11.0)	10.0 (9.0–11.0)	**<0.001** ***
Gonadotropin dose, median (IQR), IU	1250.0 (1075.0–1550.0)	1425.0 (1162.0–1775.0)	1600.0 (1350.0–2000.0)	**<0.001** ***
Estradiol level on the day of hCG trigger, median (IQR), pg/ml	3014.0 (2206.0–4300.0)	3248 (2242–4491)	3270 (2228–4648)	**0.009** **
BMI, mean (SD), kg/m^2^	20.3 (2.2)	22.4 (2.3)	25.7 (3.2)	**<0.001** ***
BMI < 18.5 kg/m^2^	224 (19.1)	33 (2.8)	3 (0.3)	**<0.001** ***
18.5 ≤ BMI < 24 kg/m^2^	899 (76.8)	878 (74.8)	349 (29.6)	
24 ≤ BMI < 28 kg/m^2^,	39 (3.3)	249 (21.2)	539 (45.8)	
BMI ≥ 28, kg/m^2^	9 (0.8)	14 (1.2)	287 (24.4)	
Systolic blood pressure, mean (SD), mmHg	117.9 (11.4)	117.9 (12.3)	120.3 (12.6)	**<0.001** ***
Diastolic blood pressure, mean (SD), mmHg	72.0 (8.0)	73.7 (8.2)	76.3 (8.8)	**<0.001** ***

Unless indicated otherwise, values are presented as No. (%) of patients.

Bold values indicate statistically significant (*P*-value < 0.05).

* *P *< 0.05;

** *P *< 0.01;

*** *P *< 0.001.

BRI, body roundness index; PCOS, polycystic ovary syndrome; AFC, antral follicle count; AMH, anti-Müllerian hormone; hCG, human chorionic gonadotropin; BMI, body mass index; SD, standard deviation; IQR, interquartile range.

### Pregnancy outcomes of IVF across BRI categories

In the overall population, the rates of live birth (53.6% for group 1, 49.5% for group 2, 45.2% for group 3, *P *< 0.001) and ongoing pregnancy (55.6% for group 1, 53.0% for group 2, 50.1% for group 3, *P *= 0.028) decreased with the increase in BRI. The total pregnancy loss rate (18.2% for group 1, 21.8% for group 2, 28.1% for group 3, *P *< 0.001) showed an increased trend with the increase in BRI (Table 2). The adjusted RRs for live birth and total pregnancy loss in highest BRI group (group 3) were 0.89 (95% CI, 0.81–0.97; *P *= 0.008) and 1.31 (95% CI, 1.07–1.61; *P *= 0.009), respectively, compared with lowest BRI group (group 1), whereas no significant associations were observed in group 2 (aRR for live birth, 0.94; 95% CI, 0.87–1.02; *P *= 0.158; aRR for total pregnancy loss, 1.10; 95% CI, 0.90–1.35; *P *= 0.356) (Table 3).

**Table 2.** Frequencies of pregnancy outcomes according to tertiles of body roundness index in the overall and BMI-stratified populations.

Outcomes	BRI group 1 (<2.54) No./total No. (%)	BRI group 2 (2.54 to <3.54) No./total No. (%)	BRI group 3 (≥3.54) No./total No. (%)	*P-*value
**Overall population (n = 3523)**				
Live birth	628/1171 (53.6)	581/1174 (49.5)	532/1178 (45.2)	**< 0.001** ***
Biochemical pregnancy	787/1171 (67.2)	775/1174 (66.0)	772/1178 (65.5)	0.677
Clinical pregnancy	697/1171 (59.5)	688/1174 (58.6)	667/1178 (56.6)	0.346
Ongoing pregnancy	651/1171 (55.6)	622/1174 (53.0)	590/1178 (50.1)	**0.028** *
Total pregnancy loss	143/787 (18.2)	169/775 (21.8)	217/772 (28.1)	**<0.001** ***
Biochemical pregnancy loss	78/787 (9.91)	65/775 (8.39)	89/772 (11.5)	0.119
Clinical pregnancy loss	65/697 (9.3)	104/697 (15.1)	128/697 (19.2)	**< 0.001** ***
**BMI 18.5 to <24.0 kg/m² (n = 2126)**				
Live birth	500/899 (55.6)	427/878 (48.6)	156/349 (44.7)	**0.001** **
Biochemical pregnancy	619/899 (68.9)	577/878 (65.7)	228/349 (65.3)	0.288
Clinical pregnancy	550/899 (61.2)	502/878 (57.2)	200/349 (57.3)	0.185
Ongoing pregnancy	515/899 (57.3)	453/878 (51.6)	166/349 (47.6)	**0.003** **
Total pregnancy loss	108/619 (17.4)	128/577 (22.2)	63/228 (27.6)	**0.004** **
Biochemical pregnancy loss	61/619 (9.9)	55/577 (9.5)	22/228 (9.6)	0.982
Clinical pregnancy loss	47/550 (8.5)	73/502 (14.5)	41/200 (20.5)	**< 0.001** ***
**BMI ≥24 kg/m² (n = 1137)**				
Live birth	28/48 (58.3)	138/263 (52.5)	374/826 (45.3)	**0.039** *
Biochemical pregnancy	31/48 (64.6)	179/263 (68.1)	542/826 (65.6)	0.746
Clinical pregnancy	29/48 (60.4)	168/263 (63.9)	465/826 (56.3)	0.090
Ongoing pregnancy	29/48 (60.4)	153/263 (58.2)	422/826 (51.1)	0.078
Total pregnancy loss	3/31 (9.7)	38/179 (21.2)	154/542 (28.4)	**0.018** *
Biochemical pregnancy loss	2/31 (6.5)	9/179 (5.0)	67/542 (12.4)	**0.011** *
Clinical pregnancy loss	1/29 (3.4)	29/168 (17.3)	87/465 (18.7)	0.111

*P*-values were obtained using the chi-square test or Fisher’s exact test, as appropriate.

Bold values indicate statistically significant (*P*-value < 0.05).

* *P *< 0.05;

** *P *< 0.01;

*** *P *< 0.001.

BRI, body roundness index; BMI, body mass index.

**Table 3.** Adjusted risk ratios of pregnancy outcomes according to tertiles of body roundness index in the overall and BMI-stratified populations.

Outcomes	BRI group 1 (<2.54)	BRI group 2 (2.54 to <3.54)		BRI group 3 (≥3.54)	
	Reference	Adjusted RR^a^ (95%CI)	*P*-value	Adjusted RR^a^ (95%CI)	*P*-value
**Overall population (n = 3523)**					
Live birth	Ref.	0.94 (0.87, 1.02)	0.158	**0.89 (0.81, 0.97)**	**0.008** **
Biochemical pregnancy	Ref.	0.98 (0.92, 1.04)	0.499	0.98 (0.92, 1.04)	0.463
Clinical pregnancy	Ref.	0.98 (0.92, 1.05)	0.605	0.95 (0.88, 1.03)	0.214
Ongoing pregnancy	Ref.	0.96 (0.89, 1.04)	0.289	0.92 (0.85, 1.00)	0.055
Total pregnancy loss	Ref.	1.10 (0.90, 1.35)	0.356	**1.31 (1.07, 1.61)**	**0.009** **
Biochemical pregnancy loss	Ref.	0.84 (0.61, 1.17)	0.310	1.13 (0.82, 1.56)	0.439
Clinical pregnancy loss	Ref.	**1.38 (1.03, 1.85)**	**0.032** *	**1.57 (1.16, 2.13)**	**0.004** **
**BMI 18.5 to <24.0 kg/m² (n = 2126)**					
Live birth	Ref.	**0.89 (0.81, 0.98)**	**0.019** *	**0.84 (0.73, 0.96)**	**0.009** **
Biochemical pregnancy	Ref.	0.95 (0.89, 1.02)	0.139	0.95 (0.86, 1.04)	0.239
Clinical pregnancy	Ref.	0.93 (0.86, 1.01)	0.087	0.94 (0.84, 1.05)	0.263
Ongoing pregnancy	Ref.	**0.91 (0.83, 0.99)**	**0.038** *	**0.85 (0.75, 0.97)**	**0.014** *
Total pregnancy loss	Ref.	1.15 (0.91, 1.47)	0.244	**1.38 (1.04, 1.85)**	**0.028** *
Biochemical pregnancy loss	Ref.	0.94 (0.65, 1.37)	0.752	0.93 (0.57, 1.51)	0.764
Clinical pregnancy loss	Ref.	**1.44 (1.01, 2.05)**	**0.047** *	**1.95 (1.28, 2.96)**	**0.002** **
**BMI ≥ 24 kg/m² (n = 1137)**					
Live birth	Ref.	0.88 (0.67, 1.15)	0.357	0.80 (0.61, 1.04)	0.089
Biochemical pregnancy	Ref.	1.02 (0.82, 1.29)	0.832	1.00 (0.80, 1.25)	0.994
Clinical pregnancy	Ref.	1.02 (0.80, 1.30)	0.886	0.89 (0.70, 1.14)	0.346
Ongoing pregnancy	Ref.	0.93 (0.72, 1.20)	0.566	0.82 (0.64, 1.06)	0.130
Total pregnancy loss	Ref.	2.13 (0.70, 6.49)	0.181	2.48 (0.82, 7.51)	0.107
Biochemical pregnancy loss	Ref.	0.88 (0.20, 3.89)	0.865	2.23 (0.56, 8.86)	0.253
Clinical pregnancy loss	Ref.	4.41 (0.64, 30.55)	0.133	3.75 (0.53, 26.52)	0.185

a Adjusted RRs were estimated using modified Poisson regression with robust variance after adjustment for age (continuous), duration of infertility (continuous), cause of infertility, type of embryo transfer, diagnosis of PCOS, number of oocytes retrieved (continuous), and number of embryo(s) transferred.

Bold values indicate statistically significant associations compared with the reference group (*P *< 0.05).

* *P *< 0.05,

** *P *< 0.01.

BRI, body roundness index; BMI, body mass index; PCOS, polycystic ovary syndrome.

### Stratified analysis by BMI category

Among women with normal BMI, the rate of live birth was lower in group 2 and group 3 (48.6% and 44.7%, respectively) compared to group 1 (55.6%), with the adjusted RRs of 0.89 (95% CI, 0.81–0.98; *P *= 0.019) and 0.84 (95% CI, 0.73–0.96; *P *= 0.009), respectively (Tables 2 and 3). Among women with normal BMI, the highest BRI group (group 3) also had a higher risk of total pregnancy loss (aRR, 1.38; 95% CI, 1.04–1.85; *P *= 0.028) compared with the lowest BRI group (group 1) (Table 3). Among women who were overweight or obese, a similar downward trend in live birth rate across BRI groups was observed (58.3%, 52.5%, and 45.3% in groups 1–3, respectively); however, the adjusted associations were not statistically significant (group 3 vs group 1: aRR, 0.80; 95% CI, 0.61–1.04; *P *= 0.089) (Table 3). The interactions between BMI category and BRI were not observed for live birth, biochemical pregnancy, clinical pregnancy, ongoing pregnancy and total pregnancy loss (all *P* for interaction > 0.05).

### The effects of BRI as continuous variables on pregnancy outcomes of IVF

**Figure 2.**
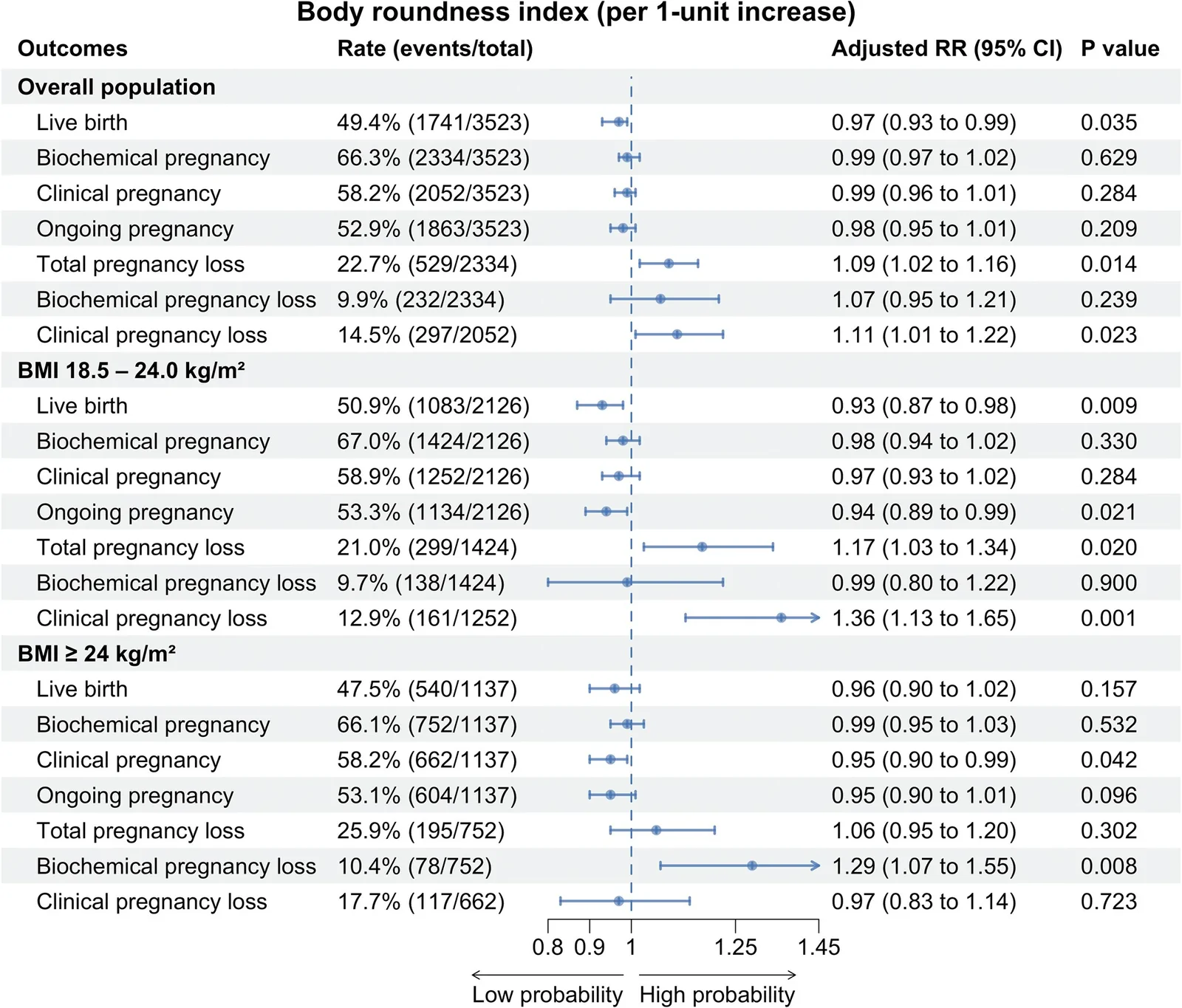
**Association of body roundness index as a continuous variable with pregnancy outcomes in the overall and BMI-stratified populations**. The forest plot shows the results of modified Poisson regression analyses examining the association between BRI (per one-unit increase) and pregnancy outcomes after IVF. Analyses were performed both in the overall population and in subgroups stratified by BMI. Adjusted factors included age (continuous), duration of infertility (continuous), cause of infertility, type of embryo transfer, diagnosis of PCOS, number of oocytes retrieved (continuous), and number of embryo(s) transferred. BRI, body roundness index; BMI, body mass index; PCOS, polycystic ovary syndrome.

The adjusted RR for live birth per one-unit increase in BRI was 0.97 (95% CI, 0.93–0.99; *P *= 0.035) in the overall population (Fig. 2). No significant associations were observed between BRI and biochemical pregnancy, clinical pregnancy or ongoing pregnancy. In the overall population, higher BRI was associated with an increased risk of total pregnancy loss (aRR per one-unit increase, 1.09; 95% CI, 1.02–1.16; *P *= 0.014) and clinical pregnancy loss (aRR per one-unit increase, 1.11; 95% CI, 1.01–1.22; *P *= 0.023). Among women with normal BMI, each one-unit increase in BRI was associated with a lower likelihood of live birth (aRR, 0.93; 95% CI, 0.87–0.98; *P *= 0.009) and ongoing pregnancy (aRR, 0.94; 95% CI, 0.89–0.99; *P *= 0.021), as well as a higher risk of total pregnancy loss (aRR, 1.17; 95% CI, 1.03–1.34; *P *= 0.020). However, these associations were not observed among women with overweight or obesity (Fig. 2). Furthermore, RCS did not show the nonlinear relationship between the BRI and pregnancy outcomes, whether in the overall population or in each BMI stratum (all *P* for nonlinearity > 0.05) (Supplementary Figs S1, S2, and S3).

### Additional analyses and sensitivity analyses

BRI was correlated with BMI in the overall population (Pearson *r* = 0.75, 95% CI 0.74–0.76; Spearman *ρ* = 0.73; both *P *< 0.001). Within BMI strata, the correlations were moderate, with Pearson r values of 0.44 (95% CI 0.41–0.48; *P *< 0.001) among women with normal BMI and 0.55 (95% CI 0.51–0.59; *P *< 0.001) among women with overweight or obesity (Supplementary Table S1). After further adjustment for BMI, the results were consistent with the main results, and collinearity diagnostics did not suggest severe multicollinearity (Supplementary Tables S2, S3, and S4).

Prespecified subgroup analyses showed no evidence of effect modification by maternal age, embryo transfer type, or tubal factor infertility (all *P* for interaction > 0.05) (Supplementary Fig. S4). In the stratified analyses by PCOS status, the associations between BRI and pregnancy outcomes of IVF were generally consistent with the main results. No significant interaction was observed between BRI and PCOS status across pregnancy outcomes for either categorical or continuous BRI (all *P* for interaction > 0.05) (Supplementary Tables S5, S6, and S7; Supplementary Figs S5, S6, and S7). For live birth, compared with the lowest BRI (group 1), the adjusted RR for the highest BRI (group 3) was 0.84 (95% CI 0.73–0.98; *P *= 0.028) among women with PCOS and 0.90 (95% CI 0.80–1.02; *P *= 0.092) among women without PCOS (Supplementary Table S5). We further performed exploratory analyses jointly stratified by PCOS status and BMI category, and these findings should be interpreted cautiously because of the smaller subgroup sizes (Supplementary Tables S6 and S7). Among women with normal BMI, the adjusted RR for the highest BRI (group 3) was 0.84 (95% CI 0.68–1.04; *P *= 0.115) among women with PCOS and 0.79 (95% CI 0.66–0.96; *P *= 0.015) among women without PCOS (Supplementary Table S6).

The results of the sensitivity analyses excluding women with underweight were generally consistent with the main results (Supplementary Tables S8 and S9). The sensitivity analyses with further adjustment for ovarian reserve and ovarian response indicators also showed generally consistent results with the main analyses (Supplementary Tables S10 and S11).

## Discussion

We examined the relationship between baseline BRI and pregnancy outcomes among women undergoing IVF. Higher BRI was associated with a lower likelihood of live birth and a higher risk of pregnancy loss compared to lower BRI. Notably, these associations were still observed among women with normal BMI. In contrast, no statistically significant associations were observed among women who were overweight or obese; however, the findings should be interpreted with caution because of limited statistical power. These findings indicate that BRI, as a complementary indicator, can identify women who have an increased risk of adverse pregnancy outcomes, despite having a normal BMI.

We found a lower rate of live birth and an increased risk of pregnancy loss in women with higher BRI compared to women with lower BRI. Few studies have evaluated the association of BRI and reproductive health. To our knowledge, no prior studies have investigated the relation between BRI and pregnancy outcomes among women undergoing IVF. Recent studies based on data from the National Health and Nutrition Examination Survey (NHANES) reported a positive association between BRI and female infertility (Wang *et al.*, 2024, 2025; Liu *et al.*, 2025). A study including 319 men undergoing infertility treatments found that higher BRI was inversely associated with semen volume (Nikolic *et al.*, 2025). In addition, two studies that compared the predictive performance of multiple obesity-related indicators in predicting female infertility found that the predictive performance of BRI was superior to BMI (Yu *et al.*, 2025a,b). Although BRI was correlated with BMI, prior studies suggested that BRI more comprehensively characterized central adiposity, which may be more closely linked to metabolic dysfunction and reproductive impairment (Müller, 2013; Mączka *et al.*, 2024; Rubino *et al.*, 2025).

Obesity may adversely affect IVF outcomes by impairing ovarian function and endometrial receptivity (Gonzalez *et al.*, 2022; Barbouni *et al.*, 2025). Several previous studies found that higher BMI was associated with impaired blastocyst formation rate, lower embryo quality, and abnormal embryo development (Comstock *et al.*, 2015; Silvestris *et al.*, 2018). In addition, central adiposity was linked to chronic low-grade inflammation and oxidative stress, which may impair endometrial receptivity and reduce the likelihood of implantation and live birth (Bellver *et al.*, 2013; Silvestris *et al.*, 2018).

Additionally, we found that higher BRI was associated with a lower likelihood of live birth even among women with normal BMI. In clinical practice, obesity is commonly assessed using BMI cutoffs. However, increasing evidence suggested that women with similar BMI may differ in body composition, fat distribution, and metabolic health (Li *et al.*, 2019). Reliance on BMI alone may therefore underestimate reproductive health risk among women with normal BMI who have excess central adiposity. Because BRI incorporates waist circumference, it can capture body shape and central adiposity, which are not fully reflected by BMI. This interpretation was supported by the moderate correlations between BRI and BMI within BMI-stratified subgroups, and the findings that the association between BRI and live birth remained consistent before and after adjustment for BMI. From a clinical perspective, BRI may complement BMI in identifying women at higher risk of adverse IVF outcomes.

One strength of this study was the use of data from multicentre randomized controlled trials with standardized outcome definitions, which significantly enhanced the generalizability and representativeness of the results. In addition, anthropometric measurements were collected by trained nurses using standardized protocols, which may reduce measurement variability and misclassification. However, several limitations should be acknowledged. First, because this was a post hoc analysis, causal inference cannot be drawn. Second, among women who were overweight or obese, the distribution across BRI categories was uneven, with only 48 women in the lowest BRI group. This small-sized subgroup may have limited statistical power and reduced the precision of effect estimates. Third, the limited number of underweight women precluded a reliable subgroup analysis, and the association between BRI and IVF outcomes in this population remains uncertain and warrants further study. Finally, although the multivariable models adjusted for several clinically relevant demographic and infertility-related factors to reduce potential confounding, residual confounding cannot be excluded, particularly unmeasured PCOS-associated metabolic features.

## Conclusion

Higher BRI was associated with a lower rate of live birth after IVF treatment, particularly among women with normal BMI. Findings among women who were overweight or obese should be interpreted with caution because of limited statistical power. Our findings suggest that BRI may serve as a complementary anthropometric measure alongside BMI for identifying women with a lower likelihood of live birth after IVF treatment. Further studies are warranted to validate these findings and clarify their clinical implications.

## Supplementary Material

hoag068_Supplementary_Data

## Data Availability

The data that support the findings of this study are available on request from the corresponding author (D.W.), upon reasonable request.
