# Supplementary material for: Association between body roundness index and pregnancy outcomes of IVF
Source: Hum Reprod Open. 2026 Aug 4;2026(3):hoag068. doi: 10.1093/hropen/hoag068 (PMC13493052; doi:10.1093/hropen/hoag068)
Supplement: Supplementary file 1 [file hoag068_supplementary_data.docx]

**Supplementary Material**

**Association between body roundness index and pregnancy outcomes of in vitro fertilization**

**1. SUPPLEMENTARY TABLES**

**Supplementary Table S1.** Correlation between continuous body roundness index and BMI.

**Supplementary Table S2.** Sensitivity analysis of pregnancy outcomes across body roundness index categories after additional adjustment for BMI.

**Supplementary Table S3.** Sensitivity analysis of pregnancy outcomes across body roundness index as a continuous variable after additional adjustment for BMI.

**Supplementary Table S4.** Collinearity diagnostics for body roundness index and BMI in BMI-adjusted sensitivity models for live birth.

**Supplementary Table S5.** Frequencies and adjusted risk ratios of pregnancy outcomes across body roundness index categories stratified by PCOS status.

**Supplementary Table S6.** Frequencies and adjusted risk ratios of pregnancy outcomes across body roundness index categories stratified by PCOS status among women with BMI 18.5 to <24.0 kg/m².

**Supplementary Table S7.** Frequencies and adjusted risk ratios of pregnancy outcomes across body roundness index categories stratified by PCOS status among women with BMI ≥ 24.0 kg/m².

**Supplementary Table S8.** Sensitivity analysis of pregnancy outcomes across body roundness index categories after excluding women with BMI <18.5 kg/m².

**Supplementary Table S9.** Sensitivity analysis of pregnancy outcomes across body roundness index as a continuous variable after excluding women with BMI <18.5 kg/m².

**Supplementary Table S10.** Sensitivity analysis of pregnancy outcomes across body roundness index categories after additional adjustment for ovarian reserve and ovarian response indicators.

**Supplementary Table S11.** Sensitivity analysis of pregnancy outcomes across body roundness index as a continuous variable after additional adjustment for ovarian reserve and ovarian response indicators.

**2. SUPPLEMENTARY FIGURES**

**Supplementary Figure S1.** Adjusted restricted cubic spline curves for the associations of body roundness index with live birth and biochemical pregnancy.

**Supplementary Figure S2.** Adjusted restricted cubic spline curves for the associations of body roundness index with clinical pregnancy and ongoing pregnancy.

**Supplementary Figure S3.** Adjusted restricted cubic spline curves for the associations of body roundness index with pregnancy loss.

**Supplementary Figure S4.** Subgroup analyses of the association between body roundness index and live birth.

**Supplementary Figure S5.** Forest plot of the association between body roundness index as a continuous variable and pregnancy outcomes stratified by PCOS status.

**Supplementary Figure S6.** Forest plot of the association between body roundness index as a continuous variable and pregnancy outcomes stratified by PCOS status among women with BMI 18.5 to <24.0 kg/m².

**Supplementary Figure S7.** Forest plot of the association between body roundness index as a continuous variable and pregnancy outcomes stratified by PCOS status among women with BMI ≥ 24.0 kg/m².

**Supplementary Table S1. Correlation between continuous** **body roundness index and BMI.**

| **Group** | **Pearson r** | **95% CI** | **Spearman ρ** |
| --- | --- | --- | --- |
| **Overall** | 0.75*** | 0.74–0.76 | 0.73*** |
| **18.5≤ BMI <24 kg/m²** | 0.44*** | 0.41–0.48 | 0.45*** |
| **BMI ≥24 kg/m²** | 0.55*** | 0.51–0.59 | 0.51*** |

Pearson and Spearman correlation coefficients were calculated using continuous BRI and continuous BMI. Women with BMI <18.5 kg/m² were excluded from the BMI-stratified analyses.

****P* < 0.001.

BMI, body mass index; CI, confidence interval.

**Supplementary Table S2. Sensitivity analysis of pregnancy outcomes across body roundness index categories after additional adjustment for BMI.**

| **Outcomes** | **BRI group 1**  **(< 2.54)** | **BRI group 2**  **(2.54 to < 3.54)** | | **BRI group 3**  **(≥ 3.54)** | |
| --- | --- | --- | --- | --- | --- |
|  | **Reference** | **Adjusted RR (95%CI)^a^** | ***P*-value** | **Adjusted RR (95%CI)^a^** | ***P*-value** |
| **Overall population (n=3523)** |  |  |  |  |  |
| Live birth | Ref. | 0.93 (0.86, 1.02) | 0.112 | **0.86 (0.77, 0.97)** | **0.011*** |
| Biochemical pregnancy | Ref. | 0.97 (0.91, 1.03) | 0.361 | 0.96 (0.88, 1.04) | 0.265 |
| Clinical pregnancy | Ref. | 0.97 (0.90, 1.05) | 0.447 | 0.93 (0.84, 1.02) | 0.133 |
| Ongoing pregnancy | Ref. | 0.94 (0.86, 1.02) | 0.112 | **0.87 (0.78, 0.96)** | **0.009**** |
| Total pregnancy loss | Ref. | 1.09 (0.88, 1.34) | 0.430 | 1.28 (0.99, 1.64) | 0.060 |
| Biochemical pregnancy loss | Ref. | 0.84 (0.59, 1.18) | 0.306 | 1.11 (0.75, 1.64) | 0.611 |
| Clinical pregnancy loss | Ref. | **1.36 (1.01, 1.84)** | **0.044*** | **1.52 (1.05, 2.19)** | **0.026*** |
| **BMI 18.5 to <24.0 kg/m² (n=2126)** |  |  |  |  |  |
| Live birth | Ref. | **0.88 (0.80, 0.98)** | **0.014*** | **0.82 (0.71, 0.95)** | **0.007**** |
| Biochemical pregnancy | Ref. | 0.93 (0.87, 1.00) | 0.054 | 0.92 (0.83, 1.01) | 0.087 |
| Clinical pregnancy | Ref. | 0.92 (0.84, 1.00) | 0.052 | 0.92 (0.82, 1.03) | 0.157 |
| Ongoing pregnancy | Ref. | **0.87 (0.78, 0.96)** | **0.009**** | **0.83 (0.72, 0.95)** | **0.006**** |
| Total pregnancy loss | Ref. | 1.13 (0.88, 1.45) | 0.357 | 1.33 (0.97, 1.82) | 0.078 |
| Biochemical pregnancy loss | Ref. | 0.91 (0.61, 1.35) | 0.641 | 0.88 (0.52, 1.47) | 0.619 |
| Clinical pregnancy loss | Ref. | 1.40 (0.97, 2.02) | 0.072 | **1.87 (1.20, 2.92)** | **0.006**** |
| **BMI ≥ 24 kg/m²(n=1137)** |  |  |  |  |  |
| Live birth | Ref. | 0.87 (0.67, 1.14) | 0.315 | 0.82 (0.62, 1.07) | 0.140 |
| Biochemical pregnancy | Ref. | 1.02 (0.81, 1.27) | 0.886 | 1.01 (0.81, 1.27) | 0.908 |
| Clinical pregnancy | Ref. | 1.01 (0.79, 1.29) | 0.954 | 0.91 (0.71, 1.16) | 0.436 |
| Ongoing pregnancy | Ref. | 0.92 (0.71, 1.18) | 0.507 | 0.84 (0.66, 1.08) | 0.183 |
| Total pregnancy loss | Ref. | 2.18 (0.71, 6.68) | 0.171 | 2.43 (0.80, 7.35) | 0.117 |
| Biochemical pregnancy loss | Ref. | 0.89 (0.20, 3.92) | 0.873 | 2.17 (0.54, 8.67) | 0.272 |
| Clinical pregnancy loss | Ref. | 4.64 (0.65, 33.06) | 0.126 | 3.70 (0.52, 26.37) | 0.192 |

^a^Adjusted RRs were estimated using modified Poisson regression with robust variance after adjustment for age (continuous), duration of infertility (continuous), cause of infertility, type of embryo transfer, diagnosis of PCOS, number of oocytes retrieved (continuous), number of embryo(s) transferred, and BMI (continuous).

Bold values indicate statistically significant associations compared with the reference group (*P* < 0.05). **P* < 0.05, ***P* < 0.01.

BRI, body roundness index; BMI, body mass index; RR, ratio risk; CI, confidence interval; PCOS, polycystic ovary syndrome.

**Supplementary Table S3. Sensitivity analysis of pregnancy outcomes across body roundness index as a continuous variable after additional adjustment for BMI.**

| **Outcomes** | **Adjusted RR^a^ (95%CI) per 1-unit increase in BRI** | ***P* value** |
| --- | --- | --- |
| **Overall population (n=3523)** |  |  |
| Live birth | **0.95 (0.91, 0.99)** | **0.044*** |
| Biochemical pregnancy | 0.98 (0.95, 1.02) | 0.321 |
| Clinical pregnancy | 0.97 (0.93, 1.01) | 0.142 |
| Ongoing pregnancy | **0.95 (0.91, 1.00)** | **0.034*** |
| Total pregnancy loss | 1.07 (0.97, 1.19) | 0.166 |
| Biochemical pregnancy loss | 1.08 (0.92, 1.28) | 0.339 |
| Clinical pregnancy loss | 1.08 (0.94, 1.24) | 0.267 |
| **BMI 18.5 to <24.0 kg/m² (n=2126)** |  |  |
| Live birth | **0.92 (0.86, 0.98)** | **0.008**** |
| Biochemical pregnancy | 0.96 (0.92, 1.01) | 0.127 |
| Clinical pregnancy | 0.96 (0.91, 1.02) | 0.182 |
| Ongoing pregnancy | **0.92 (0.86, 0.98)** | **0.010*** |
| Total pregnancy loss | 1.16 (1.00, 1.34) | 0.056 |
| Biochemical pregnancy loss | 0.96 (0.75, 1.22) | 0.744 |
| Clinical pregnancy loss | **1.34 (1.09, 1.64)** | **0.005**** |
| **BMI ≥ 24 kg/m²(n=1137)** |  |  |
| Live birth | 0.99 (0.92, 1.06) | 0.738 |
| Biochemical pregnancy | 1.01 (0.96, 1.06) | 0.801 |
| Clinical pregnancy | 0.97 (0.92, 1.03) | 0.331 |
| Ongoing pregnancy | 0.98 (0.92, 1.05) | 0.536 |
| Total pregnancy loss | 1.03 (0.89, 1.18) | 0.718 |
| Biochemical pregnancy loss | **1.30 (1.01, 1.67)** | **0.042*** |
| Clinical pregnancy loss | 0.91 (0.77, 1.09) | 0.310 |

^a^Adjusted RRs were estimated using modified Poisson regression with robust variance after adjustment for age (continuous), duration of infertility (continuous), cause of infertility, type of embryo transfer, diagnosis of PCOS, number of oocytes retrieved (continuous), number of embryo(s) transferred, and BMI (continuous).

Bold values indicate statistically significant (*P* < 0.05). **P* < 0.05, ***P* < 0.01.

BRI, body roundness index; BMI, body mass index; RR, relative ratio; CI, confidence interval; PCOS, polycystic ovary syndrome.

**Supplementary Table S4**. **Collinearity diagnostics for body roundness index and BMI in BMI-adjusted sensitivity models for live birth.**

| **Population** | **BRI** | **VIF/GVIF for BRI** | **VIF for BMI** |
| --- | --- | --- | --- |
| **Overall** | Continuous BRI | 2.61 | 2.26 |
| **18.5≤ BMI < 24 kg/m²** | Continuous BRI | 1.45 | 1.27 |
| **BMI** **≥ 24 kg/m²** | Continuous BRI | 1.70 | 1.42 |
| **Overall** | BRI Category | 1.19 | 1.33 |
| **18.5 ≤ BMI < 24 kg/m²** | BRI Category | 1.08 | 1.10 |
| **BMI ≥ 24 kg/m²** | BRI Category | 1.07 | 1.05 |

Collinearity diagnostics were performed in the sensitivity models for live birth. These models were adjusted for age (continuous), duration of infertility (continuous), cause of infertility, type of embryo transfer, diagnosis of PCOS, number of oocytes retrieved (continuous), number of embryo(s) transferred, and BMI (continuous). VIF values are presented for continuous BRI and BMI; GVIF values are presented for categorical BRI.

BRI, body roundness index; BMI, body mass index; PCOS, polycystic ovary syndrome; VIF, variance inflation factor; GVIF, generalized variance inflation factor.

**Supplementary Table S5. Frequencies and adjusted risk ratios of pregnancy outcomes across body roundness index categories stratified by PCOS status.**

| **Outcomes** | **BRI group 1**  **(< 2.54)** | **BRI group 2**  **(2.54 to < 3.54)** | **BRI group 3**  **(≥ 3.54)** | ***P* for interaction** |
| --- | --- | --- | --- | --- |
| **Live birth** |  |  |  | 0.712 |
| **PCOS** |  |  |  |  |
| Frequencies, No./total No. (%) | 110/201 (54.7) | 235/496 (47.4) | 342/774 (44.2) |  |
| Adjusted RR^a^ (95% CI); *P*-value | Reference | 0.90 (0.77, 1.05); *P*=0.191 | **0.84 (0.73, 0.98); *P*=0.028*** |  |
| **Non-PCOS** |  |  |  |  |
| Frequencies, No./total No. (%) | 518/970 (53.4) | 346/678 (51.0) | 190/404 (47.0) |  |
| Adjusted RR^a^ (95% CI); *P*-value | Reference | 0.96 (0.87, 1.05); *P*=0.372 | 0.90 (0.80, 1.02); *P*=0.092 |  |
| **Biochemical pregnancy** |  |  |  | 0.748 |
| **PCOS** |  |  |  |  |
| Frequencies, No./total No. (%) | 143/201 (71.1) | 332/496 (66.9) | 508/774 (65.6) |  |
| Adjusted RR^a^ (95% CI); *P*-value | Reference | 0.96 (0.86, 1.07); *P*=0.486 | 0.95 (0.86, 1.05); *P*=0.313 |  |
| **Non-PCOS** |  |  |  |  |
| Frequencies, No./total No. (%) | 644/970 (66.4) | 443/678 (65.3) | 264/404 (65.3) |  |
| Adjusted RR^a^ (95% CI); *P*-value | Reference | 0.98 (0.92, 1.06); *P*=0.636 | 1.00 (0.92, 1.08); *P*=0.932 |  |
| **Clinical pregnancy** |  |  |  | 0.849 |
| **PCOS** |  |  |  |  |
| Frequencies, No./total No. (%) | 127/201 (63.2) | 298/496 (60.1) | 440/774 (56.8) |  |
| Adjusted RR^a^ (95% CI); *P*-value | Reference | 0.98 (0.86, 1.11); *P*=0.731 | 0.93 (0.82, 1.05); *P*=0.257 |  |
| **Non-PCOS** |  |  |  |  |
| Frequencies, No./total No. (%) | 570/970 (58.8) | 390/678 (57.5) | 227/404 (56.2) |  |
| Adjusted RR^a^ (95% CI); *P*-value | Reference | 0.98 (0.90, 1.06); *P*=0.627 | 0.97 (0.88, 1.08); *P*=0.606 |  |
| **Ongoing pregnancy** |  |  |  | 0.845 |
| **PCOS** |  |  |  |  |
| Frequencies, No./total No. (%) | 115/201 (57.2) | 265/496 (53.4) | 384/774 (49.6) |  |
| Adjusted RR^a^ (95% CI); *P*-value | Reference | 0.96 (0.83, 1.11); *P*=0.619 | 0.90 (0.78, 1.04); *P*=0.149 |  |
| **Non-PCOS** |  |  |  |  |
| Frequencies, No./total No. (%) | 536/970 (55.3) | 357/678 (52.7) | 206/404 (51.0) |  |
| Adjusted RR* (95% CI); *P*-value | Reference | 0.95 (0.87, 1.04); *P*=0.300 | 0.94 (0.84, 1.05); *P*=0.291 |  |
| **Total pregnancy loss** |  |  |  | 0.928 |
| **PCOS** |  |  |  |  |
| Frequencies, No./total No. (%) | 32/143 (22.4) | 84/332 (25.3) | 153/508 (30.1) |  |
| Adjusted RR^a^ (95% CI); *P*-value | Reference | 1.08 (0.75, 1.54); *P*=0.684 | 1.28 (0.91, 1.79); *P*=0.152 |  |
| **Non-PCOS** |  |  |  |  |
| Frequencies, No./total No. (%) | 111/644 (17.2) | 85/443 (19.2) | 64/264 (24.2) |  |
| Adjusted RR^a^ (95% CI); *P*-value | Reference | 1.10 (0.85, 1.41); *P*=0.469 | **1.34 (1.02, 1.76); *P*=0.036*** |  |
| **Biochemical pregnancy loss** |  |  |  | 0.350 |
| **PCOS** |  |  |  |  |
| Frequencies, No./total No. (%) | 16/143 (11.2) | 23/332 (6.9) | 59/508 (11.6) |  |
| Adjusted RR^a^ (95% CI); *P*-value | Reference | 0.60 (0.33, 1.11); *P*=0.104 | 0.98 (0.58, 1.67); *P*=0.940 |  |
| **Non-PCOS** |  |  |  |  |
| Frequencies, No./total No. (%) | 62/644 (9.6) | 42/443 (9.5) | 30/264 (11.4) |  |
| Adjusted RR^a^ (95% CI); *P*-value | Reference | 0.97 (0.67, 1.40); *P*=0.857 | 1.12 (0.74, 1.69); *P*=0.598 |  |
| **Clinical pregnancy loss** |  |  |  | 0.646 |
| **PCOS** |  |  |  |  |
| Frequencies, No./total No. (%) | 16/127 (12.6) | 61/298 (20.5) | 94/440 (21.4) |  |
| Adjusted RR^a^ (95% CI); *P*-value | Reference | 1.51 (0.91, 2.50); *P*=0.109 | 1.60 (0.98, 2.62); *P*=0.060 |  |
| **Non-*P*COS** |  |  |  |  |
| Frequencies, No./total No. (%) | 49/570 (8.6) | 43/390 (11.0) | 34/227 (15.0) |  |
| Adjusted RR^a^ (95% CI); *P*-value | Reference | 1.26 (0.86, 1.86); *P*=0.236 | **1.66 (1.10, 2.52); *P*=0.017*** |  |

^a^Adjusted RRs were estimated using modified Poisson regression with robust variance after adjustment for age (continuous), duration of infertility (continuous), cause of infertility, type of embryo transfer, number of oocytes retrieved (continuous), and number of embryo(s) transferred.

Bold values indicate statistically significant associations compared with the reference group (*P* < 0.05). **P* < 0.05.

BRI, body roundness index; PCOS, polycystic ovary syndrome; RR, relative ratio; CI, confidence interval.

**Supplementary Table S6. Frequencies and adjusted risk ratios of pregnancy outcomes across body roundness index categories stratified by PCOS status among women with BMI 18.5 to <24.0 kg/m².**

| **Outcomes** | **BRI group 1**  **(< 2.54)** | **BRI group 2**  **(2.54 to < 3.54)** | **BRI group 3**  **(≥ 3.54)** | ***P* for interaction** |
| --- | --- | --- | --- | --- |
| **Live birth** |  |  |  | 0.411 |
| **PCOS** |  |  |  |  |
| Frequencies, No./total No. (%) | 80/144 (55.6) | 186/408 (45.6) | 89/194 (45.9) |  |
| Adjusted RR^a^ (95% CI); *P*-value | Reference | 0.84 (0.70, 1.00); *P*=0.056 | 0.84 (0.68, 1.04); *P*=0.115 |  |
| **Non-PCOS** |  |  |  |  |
| Frequencies, No./total No. (%) | 420/755 (55.6) | 241/470 (51.3) | 67/155 (43.2) |  |
| Adjusted RR^a^ (95% CI); *P*-value | Reference | 0.92 (0.83, 1.03); *P*=0.146 | **0.79 (0.66, 0.96); *P*=0.015*** |  |
| **Biochemical pregnancy** |  |  |  | 0.486 |
| **PCOS** |  |  |  |  |
| Frequencies, No./total No. (%) | 104/144 (72.2) | 266/408 (65.2) | 132/194 (68.0) |  |
| Adjusted RR^a^ (95% CI); *P*-value | Reference | 0.91 (0.81, 1.03); *P*=0.145 | 0.95 (0.82, 1.09); *P*=0.450 |  |
| **Non-PCOS** |  |  |  |  |
| Frequencies, No./total No. (%) | 515/755 (68.2) | 311/470 (66.2) | 96/155 (61.9) |  |
| Adjusted RR^a^ (95% CI); *P*-value | Reference | 0.97 (0.90, 1.05); *P*=0.475 | 0.92 (0.80, 1.05); *P*=0.199 |  |
| **Clinical pregnancy** |  |  |  | 0.894 |
| **PCOS** |  |  |  |  |
| Frequencies, No./total No. (%) | 91/144 (63.2) | 233/408 (57.1) | 114/194 (58.8) |  |
| Adjusted RR^a^ (95% CI); *P*-value | Reference | 0.91 (0.79, 1.06); *P*=0.244 | 0.94 (0.79, 1.11); *P*=0.463 |  |
| **Non-PCOS** |  |  |  |  |
| Frequencies, No./total No. (%) | 459/755 (60.8) | 269/470 (57.2) | 86/155 (55.5) |  |
| Adjusted RR^a^ (95% CI); *P*-value | Reference | 0.94 (0.85, 1.04); *P*=0.218 | 0.93 (0.80, 1.08); *P*=0.310 |  |
| **Ongoing pregnancy** |  |  |  | 0.929 |
| **PCOS** |  |  |  |  |
| Frequencies, No./total No. (%) | 82/144 (56.9) | 206/408 (50.5) | 93/194 (47.9) |  |
| Adjusted RR^a^ (95% CI); *P*-value | Reference | 0.90 (0.76, 1.07); *P*=0.231 | 0.86 (0.70, 1.05); *P*=0.142 |  |
| **Non-PCOS** |  |  |  |  |
| Frequencies, No./total No. (%) | 433/755 (57.4) | 247/470 (52.6) | 73/155 (47.1) |  |
| Adjusted RR^a^ (95% CI); *P*-value | Reference | 0.91 (0.82, 1.02); *P*=0.097 | **0.84 (0.70, 1.00); *P*=0.044*** |  |
| **Total pregnancy loss** |  |  |  | 0.864 |
| **PCOS** |  |  |  |  |
| Frequencies, No./total No. (%) | 23/104 (22.1) | 67/266 (25.2) | 39/132 (29.5) |  |
| Adjusted RR^a^ (95% CI); *P*-value | Reference | 1.09 (0.72, 1.64); *P*=0.680 | 1.31 (0.84, 2.04); *P*=0.242 |  |
| **Non-PCOS** |  |  |  |  |
| Frequencies, No./total No. (%) | 85/515 (16.5) | 61/311 (19.6) | 24/96 (25.0) |  |
| Adjusted RR^a^ (95% CI); *P*-value | Reference | 1.18 (0.87, 1.58); *P*=0.284 | 1.48 (1.00, 2.20); *P*=0.052 |  |
| **Biochemical pregnancy loss** |  |  |  | 0.215 |
| **PCOS** |  |  |  |  |
| Frequencies, No./total No. (%) | 13/104 (12.5) | 22/266 (8.3) | 15/132 (11.4) |  |
| Adjusted RR^a^ (95% CI); *P*-value | Reference | 0.65 (0.34, 1.25); *P*=0.197 | 0.92 (0.46, 1.87); *P*=0.827 |  |
| **Non-PCOS** |  |  |  |  |
| Frequencies, No./total No. (%) | 48/515 (9.3) | 33/311 (10.6) | 7/96 (7.3) |  |
| Adjusted RR^a^ (95% CI); *P*-value | Reference | 1.13 (0.74, 1.72); *P*=0.560 | 0.77 (0.36, 1.63); *P*=0.491 |  |
| **Clinical pregnancy loss** |  |  |  | 0.315 |
| **PCOS** |  |  |  |  |
| Frequencies, No./total No. (%) | 10/91 (11.0) | 45/233 (19.3) | 24/114 (21.1) |  |
| Adjusted RR^a^ (95% CI); *P*-value | Reference | 1.60 (0.85, 3.00); *P*=0.145 | 1.81 (0.92, 3.58); *P*=0.087 |  |
| **Non-PCOS** |  |  |  |  |
| Frequencies, No./total No. (%) | 37/459 (8.1) | 28/269 (10.4) | 17/86 (19.8) |  |
| Adjusted RR^a^ (95% CI); *P*-value | Reference | 1.26 (0.79, 2.02); *P*=0.333 | **2.39 (1.40, 4.09); *P*=0.001**** |  |

This analysis was restricted to women with BMI 18.5 to <24.0 kg/m².

^a^Adjusted RRs were estimated using modified Poisson regression with robust variance after adjustment for age (continuous), duration of infertility (continuous), cause of infertility, type of embryo transfer, number of oocytes retrieved (continuous), and number of embryo(s) transferred.

Bold values indicate statistically significant associations compared with the reference group (*P* < 0.05). **P* < 0.05, ***P* < 0.01.

BRI, body roundness index; PCOS, polycystic ovary syndrome; BMI, body mass index; RR, relative ratio; CI, confidence interval.

**Supplementary Table S7. Frequencies and adjusted risk ratios of pregnancy outcomes across body roundness index categories stratified by PCOS status among women with BMI ≥ 24.0 kg/m².**

| **Outcomes** | **BRI group 1**  **(< 2.54)** | **BRI group 2**  **(2.54 to < 3.54)** | **BRI group 3**  **(≥ 3.54)** | ***P* for interaction** |
| --- | --- | --- | --- | --- |
| **Live birth** |  |  |  | 0.535 |
| **PCOS** |  |  |  |  |
| Frequencies, No./total No. (%) | 4/6 (66.7) | 42/76 (55.3) | 253/579 (43.7) |  |
| Adjusted RR^a^ (95% CI); *P*-value | Reference | 0.81 (0.44, 1.49); *P*=0.499 | 0.66 (0.37, 1.17); *P*=0.154 |  |
| **Non-PCOS** |  |  |  |  |
| Frequencies, No./total No. (%) | 24/42 (57.1) | 96/187 (51.3) | 121/247 (49.0) |  |
| Adjusted RR^a^ (95% CI); *P*-value | Reference | 0.89 (0.66, 1.20); *P*=0.436 | 0.86 (0.64, 1.16); *P*=0.324 |  |
| **Biochemical pregnancy** |  |  |  | 0.064 |
| **PCOS** |  |  |  |  |
| Frequencies, No./total No. (%) | 5/6 (83.3) | 59/76 (77.6) | 376/579 (64.9) |  |
| Adjusted RR^a^ (95% CI); *P*-value | Reference | 0.94 (0.65, 1.37); *P*=0.755 | 0.80 (0.56, 1.15); *P*=0.226 |  |
| **Non-PCOS** |  |  |  |  |
| Frequencies, No./total No. (%) | 26/42 (61.9) | 120/187 (64.2) | 166/247 (67.2) |  |
| Adjusted RR^a^ (95% CI); *P*-value | Reference | 1.01 (0.78, 1.32); *P*=0.927 | 1.08 (0.84, 1.40); *P*=0.538 |  |
| **Clinical pregnancy** |  |  |  | 0.067 |
| **PCOS** |  |  |  |  |
| Frequencies, No./total No. (%) | 5/6 (83.3) | 58/76 (76.3) | 326/579 (56.3) |  |
| Adjusted RR^a^ (95% CI); *P*-value | Reference | 0.92 (0.64, 1.34); *P*=0.678 | **0.69 (0.49, 0.99); *P*=0.045*** |  |
| **Non-PCOS** |  |  |  |  |
| Frequencies, No./total No. (%) | 24/42 (57.1) | 110/187 (58.8) | 139/247 (56.3) |  |
| Adjusted RR^a^ (95% CI); *P*-value | Reference | 1.01 (0.76, 1.35); *P*=0.946 | 0.99 (0.74, 1.32); *P*=0.941 |  |
| **Ongoing pregnancy** |  |  |  | 0.058 |
| **PCOS** |  |  |  |  |
| Frequencies, No./total No. (%) | 5/6 (83.3) | 52/76 (68.4) | 291/579 (50.3) |  |
| Adjusted RR^a^ (95% CI); *P*-value | Reference | 0.84 (0.56, 1.24); *P*=0.380 | **0.63 (0.44, 0.91); *P*=0.014*** |  |
| **Non-PCOS** |  |  |  |  |
| Frequencies, No./total No. (%) | 24/42 (57.1) | 101/187 (54.0) | 131/247 (53.0) |  |
| Adjusted RR^a^ (95% CI); *P*-value | Reference | 0.92 (0.69, 1.24); *P*=0.595 | 0.93 (0.69, 1.24); *P*=0.609 |  |
| **Total pregnancy loss** |  |  |  | 0.496 |
| **PCOS** |  |  |  |  |
| Frequencies, No./total No. (%) | 1/5 (20.0) | 17/59 (28.8) | 114/376 (30.3) |  |
| Adjusted RR^a^ (95% CI); *P*-value | Reference | 1.47 (0.24, 9.04); *P*=0.674 | 1.50 (0.25, 8.88); *P*=0.653 |  |
| **Non-PCOS** |  |  |  |  |
| Frequencies, No./total No. (%) | 2/26 (7.7) | 21/120 (17.5) | 40/166 (24.1) |  |
| Adjusted RR^a^ (95% CI); *P*-value | Reference | 2.30 (0.58, 9.10); *P*=0.234 | 3.10 (0.80, 12.06); *P*=0.103 |  |
| **Biochemical pregnancy loss** |  |  |  | NA |
| **PCOS** |  |  |  |  |
| Frequencies, No./total No. (%) | 0/5 (0.0) | 1/59 (1.7) | 44/376 (11.7) |  |
| Adjusted RR^a^ (95% CI); *P*-value | Reference | Not estimable | Not estimable |  |
| **Non-PCOS** |  |  |  |  |
| Frequencies, No./total No. (%) | 2/26 (7.7) | 8/120 (6.7) | 23/166 (13.9) |  |
| Adjusted RR^a^ (95% CI); *P*-value | Reference | 0.90 (0.21, 3.96); *P*=0.891 | 1.78 (0.45, 7.08); *P*=0.411 |  |
| **Clinical pregnancy loss** |  |  |  | NA |
| **PCOS** |  |  |  |  |
| Frequencies, No./total No. (%) | 1/5 (20.0) | 16/58 (27.6) | 70/326 (21.5) |  |
| Adjusted RR^a^ (95% CI); *P*-value | Reference | 1.41 (0.22, 8.85); *P*=0.717 | 1.11 (0.18, 6.73); *P*=0.913 |  |
| **Non-PCOS** |  |  |  |  |
| Frequencies, No./total No. (%) | 0/24 (0.0) | 13/110 (11.8) | 17/139 (12.2) |  |
| Adjusted RR^a^ (95% CI); *P*-value | Reference | Not estimable | Not estimable |  |

This analysis was restricted to women with BMI ≥ 24.0 kg/m².

^a^Adjusted RRs were estimated using modified Poisson regression with robust variance after adjustment for age (continuous), duration of infertility (continuous), cause of infertility, type of embryo transfer, number of oocytes retrieved (continuous), and number of embryo(s) transferred.

Bold values indicate statistically significant associations compared with the reference group (*P* < 0.05). **P* < 0.05.

BRI, body roundness index; PCOS, polycystic ovary syndrome; BMI, body mass index; RR, relative ratio; CI, confidence interval.

**Supplementary Table S****8. Sensitivity analysis of pregnancy outcomes across body roundness index categories after excluding women with BMI <18.5 kg/m².**

| **Outcomes** | **BRI group 1**  **(< 2.54)** | **BRI group 2**  **(2.54 to < 3.54)** | **BRI group 3**  **(≥ 3.54)** |
| --- | --- | --- | --- |
| **Population excluding women with BMI < 18.5 kg/m^2^ (n =3263)** |  |  |  |
| **Live birth** |  |  |  |
| Frequencies, No./total No. (%) | 528/947 (55.8) | 565/1141 (49.5) | 530/1175 (45.1) |
| Adjusted RR^a^ (95% CI); *P*-value | Reference | **0.91 (0.84, 0.99); *P*=0.025*** | **0.85 (0.78, 0.94); *P*=0.001**** |
| **Biochemical pregnancy** |  |  |  |
| Frequencies, No./total No. (%) | 650/947 (68.6) | 756/1141 (66.3) | 770/1175 (65.5) |
| Adjusted RR^a^ (95% CI); *P*-value | Reference | 0.96 (0.90, 1.02); *P*=0.210 | 0.96 (0.90, 1.02); *P*=0.173 |
| **Clinical pregnancy** |  |  |  |
| Frequencies, No./total No. (%) | 579/947 (61.1) | 670/1141 (58.7) | 665/1175 (56.6) |
| Adjusted RR^a^ (95% CI); *P*-value | Reference | 0.96 (0.89, 1.03); *P*=0.221 | 0.93 (0.86, 1.00); *P*=0.056 |
| **Ongoing pregnancy** |  |  |  |
| Frequencies, No./total No. (%) | 544/947 (57.4) | 606/1141 (53.1) | 588/1175 (50.0) |
| Adjusted RR^a^ (95% CI); *P*-value | Reference | 0.93 (0.86, 1.01); *P*=0.077 | **0.89 (0.82, 0.97); *P*=0.010*** |
| **Total pregnancy loss** |  |  |  |
| Frequencies, No./total No. (%) | 111/650 (17.1) | 166/756 (22.0) | 217/770 (28.2) |
| Adjusted RR^a^ (95% CI); *P*-value | Reference | 1.17 (0.94, 1.46); *P*=0.164 | **1.39 (1.11, 1.74); *P*=0.004**** |
| **Biochemical pregnancy loss** |  |  |  |
| Frequencies, No./total No. (%) | 63/650 (9.7) | 64/756 (8.5) | 89/770 (11.6) |
| Adjusted RR^a^ (95% CI); *P*-value | Reference | 0.87 (0.62, 1.24); *P*=0.446 | 1.17 (0.83, 1.65); *P*=0.362 |
| **Clinical pregnancy loss** |  |  |  |
| Frequencies, No./total No. (%) | 48/579 (8.3) | 102/670 (15.2) | 128/665 (19.2) |
| Adjusted RR^a^ (95% CI); *P*-value | Reference | **1.54 (1.11, 2.14); *P*=0.011*** | **1.74 (1.24, 2.44); *P*=0.001**** |

Analysis included 3,263 women with BMI ≥18.5 kg/m².

^a^Adjusted RRs were estimated using modified Poisson regression with robust variance after adjustment for age (continuous), duration of infertility (continuous), cause of infertility, type of embryo transfer, diagnosis of PCOS, number of oocytes retrieved (continuous), and number of embryo(s) transferred.

Bold values indicate statistically significant associations compared with the reference group (*P* < 0.05). **P* < 0.05, ***P* < 0.01.

BRI, body roundness index; BMI, body mass index; PCOS, polycystic ovary syndrome; RR, relative ratio; CI, confidence interval.

**Supplementary Table S9. Sensitivity analysis of pregnancy outcomes across body roundness index as a continuous variable after excluding women with BMI <18.5 kg/m².**

| **Outcomes** | **Adjusted RR^a^ (95%CI) per 1-unit increase in BRI** | ***P* value** |
| --- | --- | --- |
| **Population excluding women with BMI < 18.5 kg/m^2^ (n=3263)** |  |  |
| Live birth | **0.95 (0.92, 0.99)** | **0.007**** |
| Biochemical pregnancy | 0.99 (0.96, 1.01) | 0.322 |
| Clinical pregnancy | 0.98 (0.95, 1.00) | 0.097 |
| Ongoing pregnancy | 0.97 (0.94,1.00) | 0.074 |
| Total pregnancy loss | **1.10 (1.03, 1.18)** | **0.005**** |
| Biochemical pregnancy loss | 1.10 (0.97, 1.24) | 0.144 |
| Clinical pregnancy loss | **1.13 (1.03, 1.24)** | **0.012*** |

Analysis included 3,263 women with BMI ≥18.5 kg/m².

^a^Adjusted RRs were estimated using modified Poisson regression with robust variance after adjustment for age (continuous), duration of infertility (continuous), cause of infertility, type of embryo transfer, diagnosis of PCOS, number of oocytes retrieved (continuous), and number of embryo(s) transferred.

Bold values indicate statistically significant (*P* < 0.05). **P* < 0.05, ***P* < 0.01.

BRI, body roundness index; BMI, body mass index; PCOS, polycystic ovary syndrome; RR, relative ratio; CI, confidence interval.

**Supplementary Table S10. Sensitivity analysis of pregnancy outcomes across body roundness index categories after additional adjustment for ovarian reserve and ovarian response indicators.**

| **Outcomes** | **BRI group 1**  **(< 2.54)** | **BRI group 2**  **(2.54 to < 3.54)** | | **BRI group 3**  **(≥ 3.54)** | |
| --- | --- | --- | --- | --- | --- |
|  | **Reference** | **Adjusted RR (95%CI)** | ***P*-value** | **Adjusted RR (95%CI)** | ***P*-value** |
| **Overall population (n=3523)** |  |  |  |  |  |
| **Live birth** |  |  |  |  |  |
| Model2^a^ | Ref. | 0.94 (0.87, 1.02) | 0.155 | **0.88 (0.81, 0.97)** | **0.008**** |
| Model3^b^ | Ref. | 0.95 (0.88, 1.03) | 0.235 | **0.90 (0.82, 0.99)** | **0.038*** |
| Model4^c^ | Ref. | 0.95 (0.87, 1.03) | 0.215 | **0.90 (0.81, 0.99)** | **0.031*** |
| **Biochemical pregnancy** |  |  |  |  |  |
| Model2^a^ | Ref. | 0.98 (0.92, 1.04) | 0.490 | 0.98 (0.92, 1.04) | 0.461 |
| Model3^b^ | Ref. | 0.98 (0.93, 1.05) | 0.608 | 0.99 (0.92, 1.06) | 0.697 |
| Model4^c^ | Ref. | 0.98 (0.92, 1.04) | 0.536 | 0.98 (0.92, 1.05) | 0.561 |
| **Clinical pregnancy** |  |  |  |  |  |
| Model2^a^ | Ref. | 0.98 (0.92, 1.05) | 0.601 | 0.95 (0.89, 1.03) | 0.229 |
| Model3^b^ | Ref. | 0.99 (0.92, 1.06) | 0.761 | 0.97 (0.89, 1.05) | 0.462 |
| Model4^c^ | Ref. | 0.98 (0.92, 1.06) | 0.662 | 0.96 (0.89, 1.04) | 0.338 |
| **Ongoing pregnancy** |  |  |  |  |  |
| Model2^a^ | Ref. | 0.96 (0.89, 1.04) | 0.287 | 0.92 (0.85, 1.00) | 0.059 |
| Model3^b^ | Ref. | 0.97 (0.89, 1.05) | 0.397 | 0.94 (0.86, 1.03) | 0.165 |
| Model4^c^ | Ref. | 0.96 (0.89, 1.04) | 0.342 | 0.93 (0.85, 1.02) | 0.121 |
| **Total pregnancy loss** |  |  |  |  |  |
| Model2^a^ | Ref. | 1.10 (0.90, 1.35) | 0.359 | **1.32 (1.07, 1.62)** | **0.009**** |
| Model3^b^ | Ref. | 1.10 (0.89, 1.34) | 0.386 | **1.29 (1.04, 1.60)** | **0.023*** |
| Model4^c^ | Ref. | 1.09 (0.89, 1.34) | 0.410 | **1.28 (1.03, 1.60)** | **0.027*** |
| **Biochemical pregnancy loss** |  |  |  |  |  |
| Model2^a^ | Ref. | 0.84 (0.61, 1.17) | 0.302 | 1.12 (0.81, 1.54) | 0.490 |
| Model3^b^ | Ref. | 0.83 (0.60, 1.17) | 0.289 | 1.10 (0.78, 1.55) | 0.593 |
| Model4^c^ | Ref. | 0.84 (0.60, 1.18) | 0.314 | 1.11 (0.79, 1.57) | 0.546 |
| **Clinical pregnancy loss** |  |  |  |  |  |
| Model2^a^ | Ref. | **1.38 (1.02, 1.85)** | **0.035*** | **1.58 (1.17, 2.15)** | **0.003**** |
| Model3^b^ | Ref. | **1.38 (1.02, 1.85)** | **0.034*** | **1.55 (1.12, 2.14)** | **0.007**** |
| Model4^c^ | Ref. | **1.36 (1.01, 1.82)** | **0.043*** | **1.53 (1.11, 2.10)** | **0.010*** |
| **BMI 18.5 to <24.0 kg/m² (n=2126)** |  |  |  |  |  |
| **Live birth** |  |  |  |  |  |
| Model2^a^ | Ref. | **0.89 (0.81, 0.98)** | **0.019*** | **0.84 (0.73, 0.96)** | **0.011*** |
| Model3^b^ | Ref. | **0.90 (0.82, 0.99)** | **0.029*** | **0.84 (0.73, 0.97)** | **0.014*** |
| Model4^c^ | Ref. | **0.89 (0.81, 0.98)** | **0.023*** | **0.84 (0.73, 0.96)** | **0.012*** |
| **Biochemical pregnancy** |  |  |  |  |  |
| Model2^a^ | Ref. | 0.95 (0.89, 1.02) | 0.140 | 0.95 (0.87, 1.04) | 0.261 |
| Model3^b^ | Ref. | 0.95 (0.88, 1.02) | 0.137 | 0.94 (0.86, 1.04) | 0.233 |
| Model4^c^ | Ref. | 0.94 (0.88, 1.01) | 0.110 | 0.94 (0.86, 1.03) | 0.209 |
| **Clinical pregnancy** |  |  |  |  |  |
| Model2^a^ | Ref. | 0.93 (0.86, 1.01) | 0.088 | 0.94 (0.85, 1.05) | 0.290 |
| Model3^b^ | Ref. | 0.93 (0.86, 1.02) | 0.111 | 0.94 (0.84, 1.06) | 0.312 |
| Model4^c^ | Ref. | 0.93 (0.86, 1.01) | 0.086 | 0.94 (0.84, 1.05) | 0.277 |
| **Ongoing pregnancy** |  |  |  |  |  |
| Model2^a^ | Ref. | **0.91 (0.83, 1.00)** | **0.039*** | **0.85 (0.75**–**0.97)** | **0.016*** |
| Model3^b^ | Ref. | 0.92 (0.84, 1.01) | 0.065 | **0.86 (0.75**–**0.98)** | **0.025*** |
| Model4^c^ | Ref. | 0.91 (0.83, 1.00) | 0.050 | **0.86 (0.75**–**0.98)** | **0.021*** |
| **Total pregnancy loss** |  |  |  |  |  |
| Model2^a^ | Ref. | 1.15 (0.91, 1.46) | 0.246 | **1.15 (0.91, 1.46)** | **0.029*** |
| Model3^b^ | Ref. | 1.14 (0.89, 1.45) | 0.301 | **1.37 (1.02, 1.84)** | **0.037*** |
| Model4^c^ | Ref. | 1.14 (0.89, 1.45) | 0.305 | **1.37 (1.02, 1.83)** | **0.036*** |
| **Biochemical pregnancy loss** |  |  |  |  |  |
| Model2^a^ | Ref. | 0.94 (0.65, 1.36) | 0.735 | 0.92 (0.57, 1.49) | 0.732 |
| Model3^b^ | Ref. | 0.92 (0.63, 1.35) | 0.672 | 0.90 (0.55, 1.49) | 0.691 |
| Model4^c^ | Ref. | 0.92 (0.63, 1.36) | 0.693 | 0.91 (0.55, 1.49) | 0.701 |
| **Clinical pregnancy loss** |  |  |  |  |  |
| Model2^a^ | Ref. | **1.44 (1.01, 2.05)** | **0.047*** | **1.95 (1.29, 2.97)** | **0.002**** |
| Model3^b^ | Ref. | 1.41 (0.99, 2.00) | 0.060 | **1.93 (1.26, 2.94)** | **0.002**** |
| Model4^c^ | Ref. | 1.40 (0.98, 1.99) | 0.064 | **1.93 (1.26, 2.94)** | **0.002**** |
| **BMI ≥ 24 kg/m²(n=1137)** |  |  |  |  |  |
| **Live birth** |  |  |  |  |  |
| Model2^a^ | Ref. | 0.88 (0.67, 1.15) | 0.351 | 0.79 (0.61, 1.03) | 0.084 |
| Model3^b^ | Ref. | 0.89 (0.68,1.16) | 0.375 | 0.80 (0.62, 1.05) | 0.107 |
| Model4^c^ | Ref. | 0.89 (0.68, 1.16) | 0.374 | 0.80 (0.61, 1.05) | 0.104 |
| **Biochemical pregnancy** |  |  |  |  |  |
| Model2^a^ | Ref. | 1.03 (0.82, 1.29) | 0.820 | 1.01 (0.81, 1.25) | 0.961 |
| Model3^b^ | Ref. | 1.03 (0.82, 1.29) | 0.782 | 1.02 (0.81, 1.27) | 0.888 |
| Model4^c^ | Ref. | 1.03 (0.82, 1.29) | 0.787 | 1.02 (0.82, 1.27) | 0.881 |
| **Clinical pregnancy** |  |  |  |  |  |
| Model2^a^ | Ref. | 1.02 (0.80, 1.30) | 0.875 | 0.90 (0.70, 1.14) | 0.378 |
| Model3^b^ | Ref. | 1.03 (0.80, 1.31) | 0.837 | 0.90 (0.71, 1.16) | 0.424 |
| Model4^c^ | Ref. | 1.02 (0.80, 1.31) | 0.851 | 0.90 (0.71, 1.16) | 0.422 |
| **Ongoing pregnancy** |  |  |  |  |  |
| Model2^a^ | Ref. | 0.93 (0.72, 1.20) | 0.573 | 0.83 (0.65, 1.06) | 0.142 |
| Model3^b^ | Ref. | 0.93 (0.72, 1.20) | 0.587 | 0.83 (0.65, 1.07) | 0.157 |
| Model4^c^ | Ref. | 0.93 (0.73, 1.20) | 0.583 | 0.84 (0.65, 1.07) | 0.160 |
| **Total pregnancy loss** |  |  |  |  |  |
| Model2^a^ | Ref. | 2.16 (0.71, 6.58) | 0.177 | 2.59 (0.85, 7.88) | 0.093 |
| Model3^b^ | Ref. | 2.15 (0.71, 6.53) | 0.178 | 2.51 (0.83, 7.61) | 0.102 |
| Model4^c^ | Ref. | 2.16 (0.71, 6.60) | 0.177 | 2.59 (0.85, 7.87) | 0.094 |
| **Biochemical pregnancy loss** |  |  |  |  |  |
| Model2^a^ | Ref. | 0.86 (0.19, 3.84) | 0.848 | 2.18 (0.55, 8.69) | 0.267 |
| Model3^b^ | Ref. | 0.86 (0.19, 3.85) | 0.847 | 2.24 (0.56, 8.94) | 0.252 |
| Model4^c^ | Ref. | 0.86 (0.19, 3.84) | 0.841 | 2.24 (0.56, 9.03) | 0.256 |
| **Clinical pregnancy loss** |  |  |  |  |  |
| Model2^a^ | Ref. | 4.31 (0.60, 30.84) | 0.145 | 3.96 (0.54, 28.95) | 0.175 |
| Model3^b^ | Ref. | 4.45 (0.64, 30.72) | 0.130 | 3.82 (0.54, 26.83) | 0.178 |
| Model4^c^ | Ref. | 4.31 (0.60, 30.88) | 0.145 | 3.93 (0.54, 28.60) | 0.177 |

All sensitivity models were based on the primary model (The primary model was adjusted for age (continuous), duration of infertility (continuous), cause of infertility, type of embryo transfer, diagnosis of PCOS, number of oocytes retrieved (continuous), number of embryo(s) transferred).

^a^Model 2 was additionally adjusted for AFC and AMH.

^b^Model 3 was additionally adjusted for duration of ovarian stimulation, gonadotropin dose, and estradiol level on the day of hCG trigger. ^c^Model 4 was additionally adjusted for all variables included in Models 2 and 3.

Bold values indicate statistically significant associations compared with the reference group (*P* < 0.05). **P* < 0.05, ***P* < 0.01.

BRI, body roundness index; BMI, body mass index; PCOS, polycystic ovary syndrome; RR, relative ratio; CI, confidence interval; AFC, antral follicle count; AMH, anti-Müllerian hormone; hCG, human chorionic gonadotropin.

**Supplementary Table S11. Sensitivity analysis of pregnancy outcomes across body roundness index as a continuous variable after additional adjustment for ovarian reserve and ovarian response indicators.**

| **Outcomes** | **Adjusted RR (95%CI) per 1-unit increase in BRI** | ***P* value** |
| --- | --- | --- |
| **Overall population (n=3523)** |  |  |
| **Live birth** |  |  |
| Model2^a^ | **0.96 (0.93, 1.00)** | **0.033*** |
| Model3^b^ | 0.97 (0.94, 1.01) | 0.161 |
| Model4^c^ | 0.97 (0.94, 1.01) | 0.127 |
| **Biochemical pregnancy** |  |  |
| Model2^a^ | 0.99 (0.97, 1.02) | 0.602 |
| Model3^b^ | 1.00 (0.97, 1.02) | 0.932 |
| Model4^c^ | 1.00 (0.97, 1.02) | 0.731 |
| **Clinical pregnancy** |  |  |
| Model2^a^ | 0.99 (0.96, 1.01) | 0.289 |
| Model3^b^ | 0.99 (0.96, 1.02) | 0.641 |
| Model4^c^ | 0.99 (0.96, 1.02) | 0.449 |
| **Ongoing pregnancy** |  |  |
| Model2^a^ | 0.98 (0.95, 1.01) | 0.214 |
| Model3^b^ | 0.99 (0.96, 1.02) | 0.563 |
| Model4^c^ | 0.99 (0.95, 1.02) | 0.431 |
| **Total pregnancy loss** |  |  |
| Model2^a^ | **1.09 (1.02, 1.16)** | **0.014*** |
| Model3^b^ | 1.07 (1.00, 1.16) | 0.059 |
| Model4^c^ | 1.07 (0.99, 1.15) | 0.074 |
| **Biochemical pregnancy loss** |  |  |
| Model2^a^ | 1.07 (0.95, 1.20) | 0.273 |
| Model3^b^ | 1.06 (0.93, 1.21) | 0.396 |
| Model4^c^ | 1.07 (0.93, 1.22) | 0.343 |
| **Clinical pregnancy loss** |  |  |
| Model2^a^ | **1.11 (1.02, 1.22)** | **0.021*** |
| Model3^b^ | 1.10 (0.99, 1.22) | 0.067 |
| Model4^c^ | 1.09 (0.98, 1.20) | 0.102 |
| **BMI 18.5 to <24.0 kg/m² (n=2126)** |  |  |
| **Live birth** |  |  |
| Model2^a^ | **0.93 (0.87, 0.98)** | **0.011*** |
| Model3^b^ | **0.93 (0.87, 0.99)** | **0.018*** |
| Model4^c^ | **0.93 (0.87, 0.99)** | **0.015*** |
| **Biochemical pregnancy** |  |  |
| Model2^a^ | 0.98 (0.94, 1.02) | 0.363 |
| Model3^b^ | 0.98 (0.94, 1.02) | 0.346 |
| Model4^c^ | 0.98 (0.94, 1.02) | 0.313 |
| **Clinical pregnancy** |  |  |
| Model2^a^ | 0.98 (0.93, 1.02) | 0.321 |
| Model3^b^ | 0.98 (0.93, 1.03) | 0.374 |
| Model4^c^ | 0.98 (0.93, 1.03) | 0.335 |
| **Ongoing pregnancy** |  |  |
| Model2^a^ | 0.94 (0.89, 0.99) | **0.025*** |
| Model3^b^ | 0.94 (0.89, 1.00) | **0.047*** |
| Model4^c^ | 0.94 (0.89, 1.00) | **0.040*** |
| **Total pregnancy loss** |  |  |
| Model2^a^ | 1.17 (1.03, 1.34) | **0.020*** |
| Model3^b^ | 1.17 (1.02, 1.35) | **0.028*** |
| Model4^c^ | 1.17 (1.02, 1.35) | **0.028*** |
| **Biochemical pregnancy loss** |  |  |
| Model2^a^ | 0.98 (0.79, 1.22) | 0.585 |
| Model3^b^ | 0.97 (0.78, 1.22) | 0.818 |
| Model4^c^ | 0.98 (0.78, 1.22) | 0.830 |
| **Clinical pregnancy loss** |  |  |
| Model2^a^ | 1.36 (1.13, 1.65) | **0.001**** |
| Model3^b^ | 1.36 (1.12, 1.65) | **0.002**** |
| Model4^c^ | 1.36 (1.12, 1.66) | **0.002**** |
| **BMI ≥ 24 kg/m²(n=1137)** |  |  |
| **Live birth** |  |  |
| Model2^a^ | 0.95 (0.90, 1.02) | 0.146 |
| Model3^b^ | 0.96 (0.90, 1.02) | 0.211 |
| Model4^c^ | 0.96 (0.90, 1.02) | 0.205 |
| **Biochemical pregnancy** |  |  |
| Model2^a^ | 0.99 (0.95, 1.03) | 0.567 |
| Model3^b^ | 0.99 (0.95, 1.04) | 0.753 |
| Model4^c^ | 0.99 (0.95, 1.04) | 0.727 |
| **Clinical pregnancy** |  |  |
| Model2^a^ | 0.95 (0.90, 1.00) | **0.047*** |
| Model3^b^ | 0.96 (0.91, 1.01) | 0.093 |
| Model4^c^ | 0.96 (0.91, 1.01) | 0.081 |
| **Ongoing pregnancy** |  |  |
| Model2^a^ | 0.96 (0.90, 1.01) | 0.107 |
| Model3^b^ | 0.96 (0.91, 1.02) | 0.159 |
| Model4^c^ | 0.96 (0.91, 1.02) | 0.160 |
| **Total pregnancy loss** |  |  |
| Model2^a^ | 1.08 (0.96, 1.21) | 0.219 |
| Model3^b^ | 1.07 (0.95, 1.20) | 0.274 |
| Model4^c^ | 1.07 (0.95, 1.20) | 0.250 |
| **Biochemical pregnancy loss** |  |  |
| Model2^a^ | **1.28 (1.07, 1.55)** | **0.009**** |
| Model3^b^ | **1.28 (1.06, 1.54)** | **0.010*** |
| Model4^c^ | **1.29 (1.07, 1.56)** | **0.008**** |
| **Clinical pregnancy loss** |  |  |
| Model2^a^ | 0.99 (0.85, 1.15) | 0.868 |
| Model3^b^ | 0.98 (0.84, 1.15) | 0.814 |
| Model4^c^ | 0.98 (0.84, 1.14) | 0.805 |

All sensitivity models were based on the primary model (The primary model was adjusted for age (continuous), duration of infertility (continuous), cause of infertility, type of embryo transfer, diagnosis of PCOS, number of oocytes retrieved (continuous), number of embryo(s) transferred).

^a^Model 2 was additionally adjusted for AFC and AMH.

^b^Model 3 was additionally adjusted for duration of ovarian stimulation, gonadotropin dose, and estradiol level on the day of hCG trigger.

^c^Model 4 was additionally adjusted for all variables included in Models 2 and 3.

Bold values indicate statistically significant (*P* < 0.05). **P* < 0.05, ***P* < 0.01.

BRI, body roundness index; BMI, body mass index; PCOS, polycystic ovary syndrome; RR, relative ratio; CI, confidence interval; AFC, antral follicle count; AMH, anti-Müllerian hormone; hCG, human chorionic gonadotropin.

**Supplementary Figure S1. Adjusted restricted cubic spline curves for the associations of body roundness index with live birth and biochemical pregnancy.** Restricted cubic spline curves show the associations of BRI with live birth (A, B, C) and biochemical pregnancy (D, E, F) in the overall population and according to BMI category. Solid lines indicate adjusted risk ratios, and shaded gray areas indicate 95% CIs. Restricted cubic splines were based on modified Poisson models and adjusted for age (continuous), duration of infertility (continuous), cause of infertility, type of embryo transfer, diagnosis of PCOS, number of oocytes retrieved (continuous), and number of embryo(s) transferred. BRI, body roundness index; BMI, body mass index; PCOS, polycystic ovary syndrome.

**Supplementary Figure S2.** **Adjusted restricted cubic spline curves for the associations of body roundness index with clinical pregnancy and ongoing pregnancy.** Restricted cubic spline curves show the associations of BRI with clinical pregnancy (A, B, C) and ongoing pregnancy (D, E, F) in the overall population and according to BMI category. Solid lines indicate adjusted relative ratios, and shaded gray areas indicate 95% CIs. Restricted cubic splines were based on modified Poisson models and adjusted for age (continuous), duration of infertility (continuous), cause of infertility, type of embryo transfer, diagnosis of PCOS, number of oocytes retrieved (continuous), and number of embryo(s) transferred.BRI, body roundness index; BMI, body mass index; PCOS, polycystic ovary syndrome.

**Supplementary Figure S3. Adjusted restricted cubic spline curves for the associations of body roundness index with pregnancy loss.** Restricted cubic spline curves show the associations of BRI with total pregnancy loss (A, B, C), biochemical pregnancy loss (D, E, F), and clinical pregnancy loss (G, H, I) in the overall population and according to BMI category. Solid lines indicate adjusted risk ratios, and shaded gray areas indicate 95% CIs. Restricted cubic splines were based on modified Poisson models and adjusted for age (continuous), duration of infertility (continuous), cause of infertility, type of embryo transfer, diagnosis of PCOS, number of oocytes retrieved (continuous), and number of embryo(s) transferred. BRI, body roundness index; BMI, body mass index; PCOS, polycystic ovary syndrome.

**Supplementary Figure S4. Subgroup analyses of the association between body roundness index and live birth.** The forest plot shows the results of modified Poisson regression analyses examining the association between BRI (per one unit increase) and live birth across prespecified subgroups. Subgroups included maternal age, PCOS status, embryo transfer type, and tubal factor infertility. Adjusted factors included age (continuous), duration of infertility (continuous), cause of infertility, type of embryo transfer, diagnosis of PCOS, number of oocytes retrieved (continuous), and number of embryo(s) transferred. BRI, body roundness index; PCOS, polycystic ovary syndrome; RR, relative ratio; CI, confidence interval.

**Supplementary Figure S5. Forest plot of the association between body roundness index as a continuous variable and pregnancy outcomes stratified by PCOS status.** The forest plot shows the associations between the continuous BRI and pregnancy outcomes, stratified by PCOS status. Adjusted RRs correspond to a one-unit increase in BRI. Adjusted RRs were estimated using modified Poisson regression with robust variance and adjusted for age (continuous), duration of infertility (continuous), cause of infertility, type of embryo transfer, number of oocytes retrieved (continuous), and number of embryo(s) transferred. BRI, body roundness index; RR, relative ratio; CI, confidence interval.

**Supplementary Figure S6. Forest plot of the association between body roundness index as a continuous variable and pregnancy outcomes stratified by PCOS status among women with BMI 18.5 to <24.0 kg/m².** The forest plot shows the associations between the continuous BRI and pregnancy outcomes, stratified by PCOS status, among women with BMI 18.5 to <24.0 kg/m². Adjusted RRs correspond to a one-unit increase in BRI. Adjusted RRs were estimated using modified Poisson regression with robust variance and adjusted for age (continuous), duration of infertility (continuous), cause of infertility, type of embryo transfer, number of oocytes retrieved (continuous), and number of embryo(s) transferred. BRI, body roundness index; PCOS, polycystic ovary syndrome; BMI, body mass index; RR, relative ratio; CI, confidence interval.

**Supplementary Figure S7. Forest plot of the association between body roundness index as a continuous variable and pregnancy outcomes stratified by PCOS status among women with BMI ≥ 24.0 kg/m².** The forest plot shows the associations between the continuous BRI and pregnancy outcomes, stratified by PCOS status, among women with BMI ≥ 24.0 kg/m². Adjusted RRs correspond to a one-unit increase in BRI. Adjusted RRs were estimated using modified Poisson regression with robust variance and adjusted for age (continuous), duration of infertility (continuous), cause of infertility, type of embryo transfer, number of oocytes retrieved (continuous), and number of embryo(s) transferred. BRI, body roundness index; PCOS, polycystic ovary syndrome; BMI, body mass index; RR, relative ratio; CI, confidence interval.
